# A slow-cycling/quiescent cells subpopulation is involved in glioma invasiveness

**DOI:** 10.1038/s41467-022-32448-0

**Published:** 2022-08-15

**Authors:** Francesco Antonica, Lucia Santomaso, Davide Pernici, Linda Petrucci, Giuseppe Aiello, Alessandro Cutarelli, Luciano Conti, Alessandro Romanel, Evelina Miele, Toma Tebaldi, Luca Tiberi

**Affiliations:** 1 Armenise-Harvard Laboratory of Brain Cancer, Department CIBIO, University of Trento, 38123 Trento, Italy; 2 Laboratory of Stem Cell Biology, Department CIBIO, University of Trento, 38123 Trento, Italy; 3 Laboratory of Bioinformatics and Computational Genomics, Department CIBIO, University of Trento, 38123 Trento, Italy; 4 Department of Paediatric Haematology/Oncology, Cell and Gene Therapy, Bambino Gesù Children’s Hospital, IRCCS, Rome, Italy; 5 Laboratory of RNA and Disease Data Science, Department CIBIO, University of Trento, 38123 Trento, Italy; 6 Section of Hematology, Yale Cancer Center and Department of Internal Medicine, Yale School of Medicine, 06511 New Haven, US

**Keywords:** CNS cancer, Cancer stem cells, Cancer models

## Abstract

Pediatric and adult high-grade gliomas are the most common primary malignant brain tumors, with poor prognosis due to recurrence and tumor infiltration after therapy. Quiescent cells have been implicated in tumor recurrence and treatment resistance, but their direct visualization and targeting remain challenging, precluding their mechanistic study. Here, we identify a population of malignant cells expressing Prominin-1 in a non-proliferating state in pediatric high-grade glioma patients. Using a genetic tool to visualize and ablate quiescent cells in mouse brain cancer and human cancer organoids, we reveal their localization at both the core and the edge of the tumors, and we demonstrate that quiescent cells are involved in infiltration of brain cancer cells. Finally, we find that Harmine, a DYRK1A/B inhibitor, partially decreases the number of quiescent and infiltrating cancer cells. Our data point to a subpopulation of quiescent cells as partially responsible of tumor invasiveness, one of the major causes of brain cancer morbidity.

## Abstract (web summary)

Quiescent cancer stem cells have been particularly associated to chemoresistance. Here, the authors show that a slowcycling subpopulation in high-grade glioma patients can invade the brain to promote tumourigenesis.

## Introduction

Pediatric and adult high-grade gliomas are the most common primary malignant brain tumors, with a median survival of just a few years. Conventional therapies, including surgery, radiotherapy, and pharmacotherapy (typically chemotherapy with temozolomide), have not resulted in major improvements in the survival outcomes of patients with high-grade gliomas^1^. Reasons for this lack of progress include invasive tumor growth in the surrounding brain, precluding curative surgical resection and limiting the utility of local therapy. Glioblastoma (GBM) cancer stem cells have been proposed to be involved in this process but how high-grade gliomas infiltration occurs is still poorly understood^2,3^. The existence of quiescent/slow-cycling stem cells in brain cancer is mainly supported by scRNAseq, mathematical modeling of xenograft outgrowth assays and mouse models, but their specific/direct visualization and targeting remain evasive^4–7^. Lineage tracing assays based on genetic mouse models have proposed that quiescent/slow-cycling cells could promote brain tumor recurrence following chemotherapy^3,4,7–9^, but their role in glioma infiltration remains unknown. Furthermore, because of their lower rate of proliferation, quiescent/slow-cycling cells are resistant to classical anticancer drugs^2^. Therefore, there is a major need of novel therapeutical approaches to target quiescent cells in pediatric and adult high-grade gliomas. In this work, we use multi-disciplinary approaches to specifically investigate the quiescent cancer cells highlighting their involvement during the invasion of high-grade glioma.

## Results

### Characterization of a population of malignant non-cycling PROM1^+^ cells in human glioblastoma

**Fig. 1:**
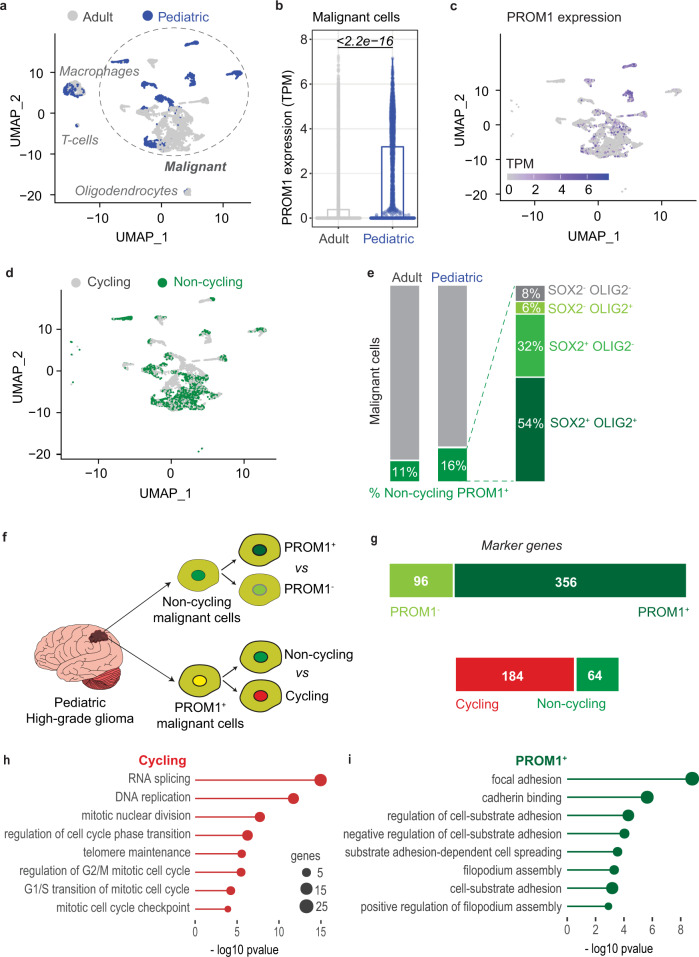
Characterization of a population of non-cycling PROM1^+^ cells in pediatric glioblastomas. **a** UMAP representation 7930 cells, profiled with single cell RNA-seq from 28 glioblastomas in Ref. 15. Malignant cells are highlighted. Cells from adult and pediatric glioblastomas, 5742 and 2188 respectively, are color-coded. **b** Distributions of PROM1 expression in adult and pediatric glioblastoma malignant cells (*n* = 4916 adult malignant cells; *n* = 1947 pediatric malignant cells). Differences in the distributions were tested with two-tailed Wilcoxon rank-sum test. **c** UMAP representation of glioblastoma malignant cells, color-coded according to PROM1 expression. **d** UMAP representation of cycling and non-cycling glioblastoma malignant cells (3671 and 3192, respectively). **e** Percentage of non-cycling PROM1^+^ cells among malignant cells of adult and pediatric glioblastomas. Pediatric non-cycling PROM1^+^ cells were further classified according to the expression of SOX2 and OLIG2 into four subpopulations. **f** Schematic representations of the comparisons performed to characterize specific subpopulations of malignant cells from pediatric glioblastomas: among non-cycling cells, 313 PROM1^+^ cells were compared to 548 PROM1^-^ cells (PROM1^+^ vs PROM1^-^). Among PROM1^+^ cells, 313 non-cycling and 523 cycling cells were compared (non-cycling vs cycling). **g** Number of marker genes identified by comparing non-cycling PROM1^+^ vs PROM1^-^ cells (top bar) and cycling vs non-cycling PROM1+ cells (bottom bar). **h** Gene Ontology enrichment analysis of marker genes more expressed in cycling PROM1^+^ cells when compared to non-cycling cells (*n* = 313 non-cycling pediatric PROM1+ malignant cells; *n* = 523 cycling pediatric PROM1+ malignant cells) (one-sided Fisher’s exact test). **i** Gene Ontology enrichment analysis of marker genes more expressed in non-cycling PROM1^+^ cells when compared to PROM1^-^ cells (*n* = 313 non-cycling pediatric PROM1+ malignant cells; *n* = 548 non-cycling pediatric PROM1- malignant cells) (one-sided Fisher’s exact test).

Prominin-1 (PROM1; also called CD133) is a marker of cancer stem cells in many types of cancer, including human brain cancer. Prominin-1 positive (PROM1^+^) cells are considered tumor-initiating cells^10^ characterized by resistance to radiation^11^ and chemotherapy^12^ and thought to be involved in cancer relapse^13^. Furthermore, quiescent PROM1^+^ cells have been also shown to be present in normal mouse brain^14^, but few data are available on human brain cancer samples. Here, we sought to investigate the presence of a quiescent PROM1^+^ subpopulation in samples of adult and pediatric glioblastomas. Notably, GBM is a highly heterogenous cancer, as recently demonstrated by Neftel and colleagues by performing single-cell RNA sequencing of 20 adult and 8 pediatric glioblastomas^15^. To address whether a population of PROM1^+^ cells with quiescent feature exist in human brain cancer, we reanalyzed the single cell dataset^15^, consisting of the expression profiles of 7930 high-quality cells, 5742 from adult and 2188 from pediatric tumors (Fig. 1a). Among these cells, 6863 were defined as malignant based on the presence of chromosomal copy number aberrations^15^ (Fig. 1a). By measuring the expression of PROM1 in malignant cells, we observed higher levels in cells from pediatric glioblastomas (Fig. 1b, c). Based on the expression levels of known cell-cycle related genes, in particular two modules associated with G1/S and G2/M phases (Supplementary Fig. 1a–c, Methods), malignant cells were further classified into cycling and non-cycling (including quiescent cells), 3671 and 3192 respectively (Fig. 1d). Combining this classification with the expression of PROM1, we identified a population of non-cycling PROM1^+^ cells, amounting to 11% of adult and 16% (313 cells) of pediatric malignant glioblastoma cells (Fig. 1e). Pediatric non-cycling PROM1^+^ cells were further classified according to the expression of other putative glioma stem cells marker such as SOX2 and OLIG2: 54% expressed both markers, 32% only SOX2, 6% only OLIG2, and 8% neither (Fig. 1e, right). To further characterize the population of 313 malignant non-cycling PROM1^+^ cells from pediatric glioblastomas, we compared their expression levels with 548 non-cycling PROM1^-^ cells (Fig. 1f, upper panel, Supplementary Data 1), and with 523 cycling PROM1^+^ cells (Fig. 1f lower panel, Supplementary Data 2) and we identified specific sets of marker genes (Fig. 1g). Cycling PROM1^+^ cells, when compared to non-cycling cells, displayed higher expression of genes related with cell cycle programs (Fig. 1h). Importantly, non-cycling PROM1^+^ cells, compared to PROM1^-^ cells, were characterized by high expression of genes associated with cell adhesion and spreading programs (Fig. 1i). Overall, we identified a population of malignant non-cycling PROM1^+^ cells (including quiescent cells), consistently present in pediatric brain tumors.

### In vivo visualization of quiescent cells in mouse glioma model

**Fig. 2:**
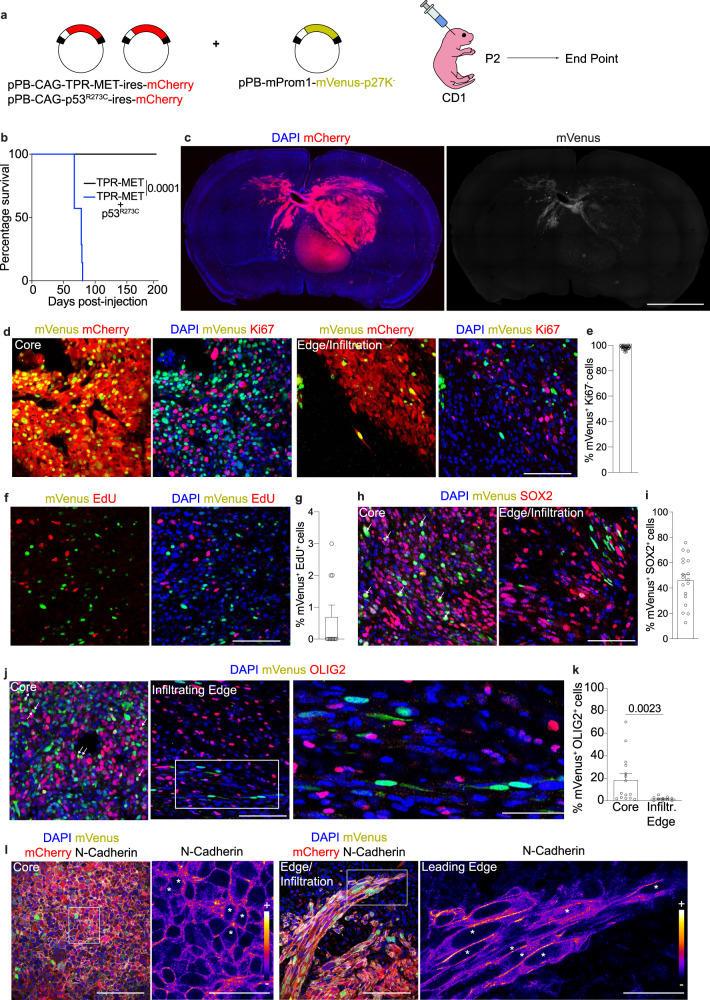
Direct visualization of quiescent Prom1^+^ cells in the brain tumor of mice. **a** Electroporation of cells in the SVZ of P2 CD1 mice with pPB-CAG-TPR-MET-ires-mCherry and pPB-CAG-p53^R273C^-ires-mCherry (TP-Cherry) together with pPB-mProm1-mVenus-p27K^−^ (Prom1-Venus-p27). Mice were sacrificed at humane endpoint. **b** Survival curve (*p* = 0.0001) of mice injected with only TPR-MET (*n* = 7 mice, black line) or both TPR-MET and p53^R273C^ (*n* = 7 mice, blue line). **c** Mosaic images of brain sections showing Prom1-Venus-p27 expressing cells within the tumor (mCherry^+^) (*n* = 10 mice). **d**, **e** Images (**d**) and quantifications (**e**) of mVenus^+^ cells (green) not expressing Ki67 (red) in the core and edge/infiltrative regions of the mCherry^+^ tumors expressing Prom1-Venus-p27 (*n* = 4 mice, 4818 cells). Each dot represents a brain section. **f**, **g** Images (**f**) and quantifications (**g**) of mVenus^+^ cells (green) labeled with EdU (red) (*n* = 2 mice, 429 cells). Cells in the SVZ of P2 CD1 mice were electroporated with pPB-CAG-TPR-MET-ires-mCherry and pPB-CAG-p53^R273C^-ires-mCherry (TP-Cherry) together with pPB-mProm1-mVenus-p27K^−^ (Prom1-Venus-p27). Mice were injected with EdU at P44 and brains were dissected at P45. **h**, **i** Images (**h**) and quantifications (**i**) of mVenus^+^ cells (green) expressing SOX2 (red) in the core and edge/infiltrative regions of the mCherry^+^ tumors expressing Prom1-Venus-p27 (*n* = 3 mice, 1966 cells). **j**, **k** Images (**j**) and quantifications (**k**) of mVenus^+^ cells (green) expressing OLIG2 (red) in the core and edge/infiltrative regions of the mCherry^+^ tumors expressing Prom1-Venus-p27 (for core *n* = 4 mice, 3753 cells; for infiltrating edge *n* = 4 mice, 1104 cells). **l** Images of mVenus^+^ cells (green) co-expressing N-cadherin (gray) in the core or edge/infiltrative areas of the mCherry^+^ tumors (red) expressing Prom1-Venus-p27 (*n* = 6 mice). N-cadherin expression level is shown as multi-colored LUT fire. Asterisks refer to Venus-p27 expressing cells. Scale bars (**c**) 2 mm, (**d**, **f**, **h**, **j**, **l**) 100 μm, (**l**, ROI) 50 μm, (**j**, ROI) 40 μm. Data are presented as mean ± SEM. P values are calculated by unpaired two-tailed non-parametric Mann–Whitney test (**k**). Kaplan–Meier survival curve followed by Log-rank (Mantel–Cox) test was used for testing difference in the survival of mice (**b**). Source data are provided as a Source Data file.

To highlight the possible role of quiescent PROM1^+^ cells in brain tumor we decided to develop an approach to visualize them first and then proceed to a further characterization. To directly visualize quiescent cells in vivo we took advantage of the G0 fluorescent reporter mVenus-p27K^−^^16–18^. The reporter consists of a fluorescent protein (mVenus) fused to a mutant form of p27 (p27K^−^) that leads to mVenus degradation in proliferating cells^16,19^. We first validated the system in neural stem cells (NSCs) of normal mouse brain where mVenus-p27K^−^ (Venus-p27) expression was controlled by the promoters of two genes expressed in quiescent stem cells: mouse *Prom1* promoter^14^ (Prom1-Venus-p27) or human *SOX2* promoter^20^ (Sox2-Venus-p27) (Supplementary Fig. 2a, b). Afterwards, we confirmed the specificity of mouse *Prom1* promoter by electroporating the cells in the subventricular zone (SVZ) of newborn *Prom1*-CreERT2 knock-in mice with two vectors expressing mVenus under the control of CreERT2 activity and mCherry expression under the control of Prom1 promoter, respectively (Supplementary Fig. 2c–e). We also confirmed the specificity of the human *SOX2* minimal promoter in targeting SOX2^+^ cells (Supplementary Fig. 2f, g). We next checked whether the mVenus-p27K^−^ biosensor labels quiescent Prom1^+^ or Sox2^+^ cells in the SVZ of mouse brain (Supplementary Fig. 3a). First, we found the absence of the proliferation marker Ki67 in 98.2% and 98.7% of Prom1-Venus-p27^+^ (qProm1) or Sox2-Venus-p27^+^(qSox2) cells (Supplementary Fig. 3b, c), respectively. Because cells in early G1 might not express high level of Ki67, we confirmed that qProm1 or qSox2 cells respectively did not incorporate 5-ethynyl-2′-deoxyuridine (EdU) when injected 24 h (Supplementary Fig. 3d-f) or 7 days (Supplementary Fig. 3g-i) prior to sacrifice. These data indicate that our system labels quiescent/slow-cycling cells in the mouse brain. We next used the same approach to visualize qProm1 and qSox2 cells within brain tumors. To do so, brain cancer was induced by overexpression of a ligand-independent form of human *MET* (TPR-MET) and mutant *TP53*^21,22^ (Fig. 2a). We found that the combination of both TPR-MET and p53^R273C^ (TP-Cherry) led to the formation of highly aggressive and lethal tumors (Fig. 2b, c). This was confirmed by RNA-seq analysis, showing high similarity to the transcriptomes of published mouse brain tumors^23^ (Supplementary Fig. 4a). The co-expression of Prom1-Venus-p27 revealed the presence of qProm1 cells across the whole tumor as in tumor core and infiltrating edge (Fig. 2c, d). Firstly, we found that the marker of proliferation Ki67 was not detected in 98.3% of qProm1 (Fig. 2d, e). Similarly, 99.8% of qSox2 cells were negative for Ki67 (Supplementary Fig. 4b–d). Furthermore, we confirmed that only 0.7% of qProm1 cells incorporated EdU within 24 h pulse period (Fig. 2f, g). Afterwards, we confirmed that qProm1 (Fig. 2h–k) and also qSox2 (Supplementary Fig. 4e–h) cells expressed two non-specific glioma stem cell (GSC) markers such as SOX2^24^ and OLIG2^25,26^. We observed that about half of qProm1 cells express also SOX2, suggesting the possible co-existence of two different quiescent populations, Prom1^+^/SOX2^+^ and Prom1^+^/SOX2^-^ (Fig. 2h, i), as also observed in human brain tumor samples (Fig. 1e). qProm1 (Fig. 2j, k) cells rarely expressed OLIG2 in the infiltrating edge. The analysis of N-Cadherin expression together with the observation of cell shape suggests a mesenchymal-like morphology of qProm1 cells located in the infiltrating edge (Fig. 2l, Supplementary Movie 1). Indeed, qProm1 cells showed a typical epithelial cobblestone morphology in the core, while they adopted an elongated shape with a strong accumulation of N-cadherin at the cell-cell contact in the infiltrating edge (Fig. 2l). These data confirm the presence of quiescent GSCs across different regions of brain tumor^3–5,7^ and, more importantly, they indicate their localization not only in the tumor core, but also in the infiltrating edge, in accordance with Liu and colleagues, that found a putative quiescent stem cell population at the human and mouse brain tumor border^3^.

### In vivo lineage tracing of qProm1 glioma stem cells

**Fig. 3:**
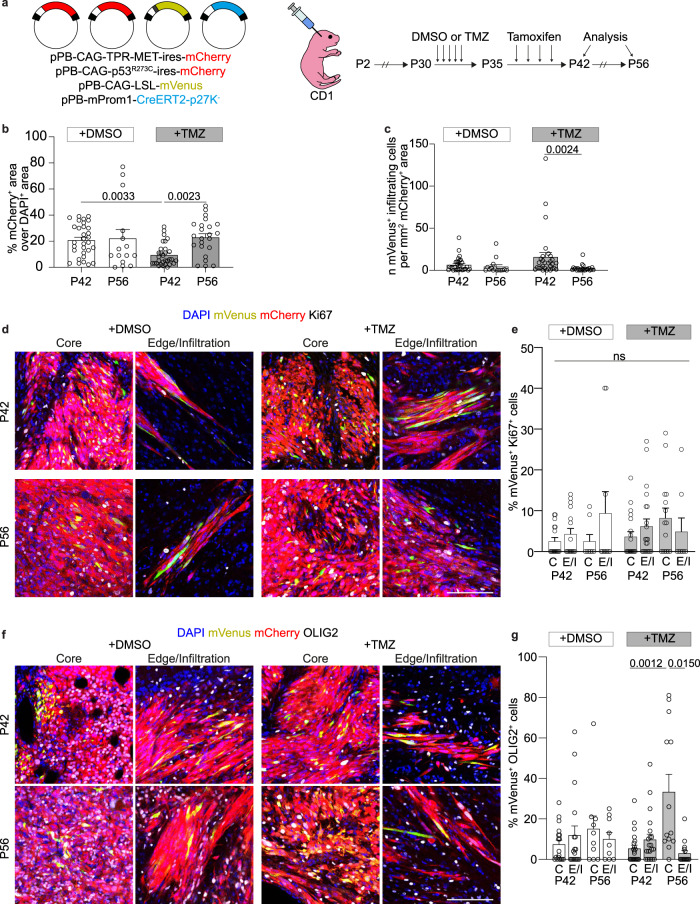
Lineage tracing of quiescent Prom1^+^ cells in TP-induced tumors after Temozolomide treatment. **a** Electroporation of cells in the SVZ of P2 CD1 mice with pPB-CAG-TPR-MET-ires-mCherry and pPB-CAG-p53^R273C^-ires-mCherry together with pPB-CAG-LSL-mVenus and pPB-mProm1-CreERT2-p27K^−^. Mice were injected with DMSO or Temozolomide (TMZ) every day from P30 to P34 and then with Tamoxifen every two days from P35 to P41. Brains were dissected at P42 and P56. **b** Quantification of the tumor size shown as percentage of mCherry^+^ area over DAPI^+^ area in brain sections of DMSO- (*n* = 7 mice at P42; *n* = 3 mice at P56) and TMZ-treated (*n* = 7 mice at P42; *n* = 5 mice at P56). Each dot represents a brain section. **c** Quantification of mVenus^+^ infiltrating cells in the tumor of DMSO- (*n* = 7 mice at P42; *n* = 3 mice at P56) and TMZ-treated (*n* = 7 mice at P42; *n* = 5 mice at P56). Each dot represents a brain section. **d**, **e** Images (**d**) and quantifications (**e**) of mVenus^+^ cells (green) co-expressing Ki67 (gray) in the core or edge/infiltrative regions of the mCherry^+^ tumors (red) of DMSO- (*n* = 6 mice at P42, 576 cells for core and 380 cells for infiltrating edge; *n* = 3 mice at P56, 219 cells for core and 85 cells for infiltrating edge) and TMZ-treated (*n* = 7 mice at P42, 613 cells for core and 1058 cells for infiltrating edge; *n* = 5 mice at P56, 952 cells for core and 82 cells for infiltrating edge). Each dot represents a tumor area. **f**, **g** Images (**f**) and quantifications (**g**) of mVenus^+^ cells (green) co-expressing OLIG2 (gray) in the core or edge/infiltrative areas of the mCherry^+^ tumors (red) of DMSO- (*n* = 6 mice at P42, 736 cells for core and 447 cells for infiltrating edge; *n* = 3 mice at P56, 237 cells for core and 123 cells for infiltrating edge) and TMZ-treated (*n* = 7 mice at P42, 722 cells for core and 527 cells for infiltrating edge; *n* = 5 mice at P56, 1620 cells for core and 229 cells for infiltrating edge). Scale bars (**d**, **f**) 100 μm. Data are presented as mean ± SEM. *p* values calculated by Kruskal–Wallis test followed by Dunn’s test for multiple comparison (**b**, **c**, **d**). Source data are provided as a Source Data file.

The major challenge in performing genetic lineage tracing of quiescent cancer stem cells is to specifically trace non-proliferating cells and to develop a tool that can be used for different human tumors. Here, we developed an inducible Cre recombinase (CreERT2) fused with p27K^−^ (CreERT2-p27K^−^), under the control of the *Prom1* promoter. The specificity of the approach was confirmed by EdU incorporation demonstrating that our system labels cell progeny of quiescent cells (Supplementary Fig. 5a–c); moreover following lineage tracing 96.5% of qProm1-derived cells were positive for SOX2 in normal mouse SVZ (Supplementary Fig. 5d–f). We next sought to use this system to trace the qProm1 progeny cells in TP-induced tumors, with and without chemotherapy treatment. Mice were treated with Temozolomide (TMZ) for 5 days followed by 7 days labeling of qProm1 cells with Tamoxifen (Tam) (Fig. 3a). Firstly, we confirmed lack of mVenus^+^ cells in absence of Tamoxifen (Supplementary Fig. 6a). To obtain an unbiased characterization, we analyzed the whole brain section, measuring the areas of brain (DAPI^+^) and tumor (mCherry^+^) (Fig. 3b and Supplementary Fig. 6b). As expected, we confirmed the efficacy of TMZ treatment on reducing tumor size, as well as the consequent fast relapse after chemotherapy administration (Fig. 3b). Since the cell ability to infiltrate is one of the deadliest features of high-grade glioma, we analyzed the number of infiltrating qProm1-derived progeny. In TMZ-treated mice, we did not observe significant differences with respect to the DMSO control group (Fig. 3c). Afterwards, we analyzed the proliferation marker Ki67 in qProm1-derived cells in the core or edge/infiltration regions of tumors at P42 and P56. Interestingly, we did not observe any significant difference between DMSO or TMZ treatments (Fig. 3d, e and Supplementary Fig. 6c). Finally, considering that the majority of qProm-1 showed a lower rate of proliferation, we checked whether mVenus^+^ cells were positive also for markers of neurons (NeuN) or stem/progenitors (SOX2). We rarely observed Venus^+^/NeuN^+^ and, as expected, they were mainly Ki67^-^ (Supplementary Fig. 6d, e). Furthermore, when we analyzed the proliferation rate of Venus^+^/Sox2^+^ cells, we observed they were mostly Ki67- with no difference with respect to both DMSO or TMZ groups (Supplementary Fig. 6f, g). We then analyzed the distribution of the glioma stem cell marker OLIG2 (Fig. 3f) observing a statistically significant increase only in the core of TMZ-treated mice over time (Fig. 3g). Overall, these data suggest that TMZ treatment does not have a major effect on qProm1 activation and it does not affect proliferation of their progeny.

### In vivo lineage ablation of quiescent glioma stem cells

**Fig. 4:**
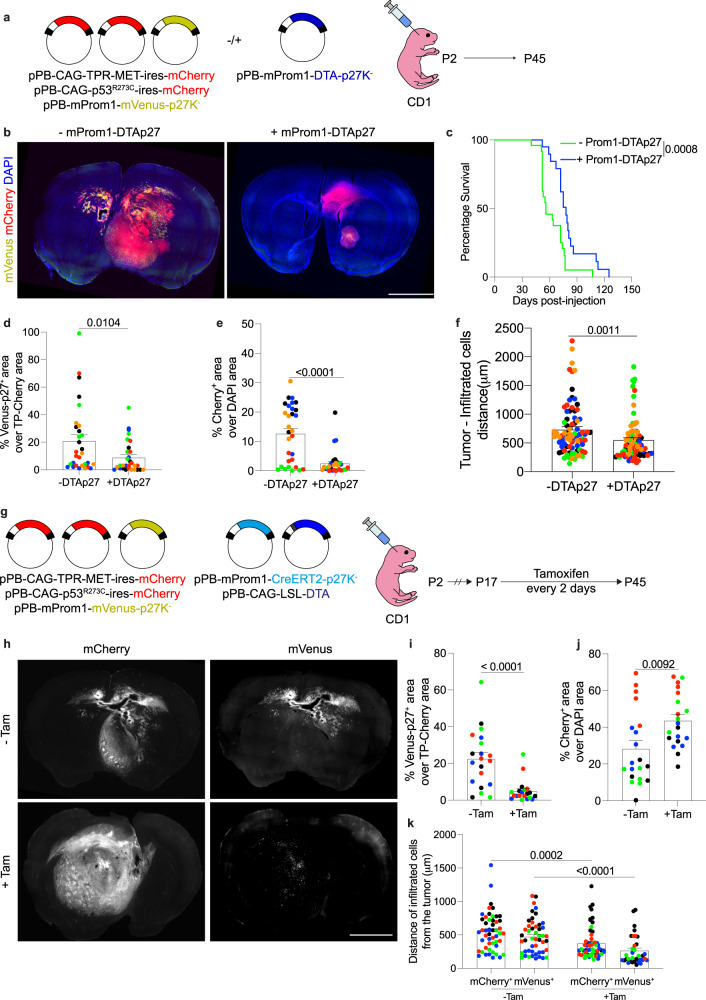
Ablation of quiescent Prom1^+^ cells reduces infiltration in TP-induced tumors. **a** Electroporation of cells in the SVZ of P2 CD1 mice with pPB-CAG-TPR-MET-ires-mCherry, pPB-CAG-p53^R273C^-ires-mCherry and pPB-mProm1-mVenus-p27K^−^ with or without pPB-mProm1-DTA-p27K^−^. **b** Images of brain sections showing Prom1-Venus-p27 expressing cells within the tumor (mCherry^+^) of mice co-electroporated without (*n* = 5 mice) or with (*n* = 5 mice) pPB-mProm1-DTA-p27K^−^ (Prom1-DTA-p27). **c** Survival curve of mice injected with pPB-CAG-TPR-MET-ires-mCherry, pPB-CAG-p53^R273C^-ires-mCherry and pPB-mProm1-mVenus-p27K^−^ without (*n* = 23 mice) or with pPB-mProm1-DTA-p27K^−^ (*n* = 18 mice). **d**, **e** Quantifications of mVenus^+^ (green) (**d**) and mCherry^+^ (red) (**e**) area in the brain sections of mice co-electroporated without (*n* = 5 mice) or with (*n* = 5 mice) Prom1-DTA-p27. Each dot represents a brain section and color code is applied to each mouse. **f** Quantification of the distance of the infiltrated mCherry^+^ cells from the tumor edge in the brain sections of mice co-electroporated without (*n* = 5 mice) or with (*n* = 5 mice) Prom1-DTA-p27. The furthest 3 cells for each section were considered; each dot represents a cell and color code is applied to each mouse. **g** Electroporation of cells in the SVZ of P2 CD1 mice with pPB-CAG-TPR-MET-ires-mCherry, pPB-CAG-p53^R273C^-ires-mCherry, pPB-mProm1-mVenus-p27K^−^, pPB-mProm1-CreERT2-p27K^−^ and pPB-CAG-LSL-DTA. Mice were injected with Tamoxifen every two days from P17 to P45. **h**–**j**, Images (**h**) and quantifications of mVenus^+^ (green) (**i**) and mCherry^+^ (red) (**j**) area in the brain sections of mice un-injected (*n* = 4) or injected (*n* = 4 mice) with Tamoxifen (Tam). Each dot represents a brain section and color code is applied to each mouse. **k** Quantification of the distance of the infiltrated mCherry^+^ or mVenus^+^ cells from the tumor edge in the brain sections of mice un-injected (*n* = 4 mice) or injected (*n* = 4 mice) with Tamoxifen. The furthest 3 cells for each section were considered; each dot represents a cell and color code is applied to each mouse. Scale bars (**b**, **h**) 2 mm. Data are presented as mean ± SEM. P values were calculated by unpaired two-tailed non-parametric Mann–Whitney test (**d**, **e**, **f**, **i**, **k**) or unpaired two-tailed Student’s *t* test (**j**). Kaplan–Meier survival curve followed by Log-rank (Mantel-Cox) test was used for testing difference in the survival of mice (**b**). Source data are provided as a Source Data file.

We next sought to investigate the role of the qProm1 cells in tumorigenesis by ablating them, using diphtheria toxin fragment A (DTA) fused to p27K^−^ isoform (DTA-p27). The efficiency of ablation was verified in newborn SVZ with DTA-p27 (Supplementary Fig. 7a), revealing the absence of qProm1 cells (Supplementary Fig. 7b, c). We also validated DTA-mediated ablation in qSox2 cells (Supplementary Fig. 7d-f). Afterwards we checked the presence of qProm1 cells in TP-induced tumors in absence or presence of DTA-p27 (Fig. 4a). Mice were sacrificed at P45 and brain sections were histologically analyzed (Fig. 4b). Compared to control mice, the co-injection of DTA-p27 led to an overall increased survival (median survival days: 56 for control vs 78 for +Prom1-DTA-p27 group) (Fig. 4c). Furthermore, a reduced number of qProm1 cells (Fig. 4d) was accompanied with a smaller tumor size (Fig. 4e). Interestingly, we also observed a strong reduction of tumor infiltrated cells (Fig. 4f). To investigate the effect of the depletion of qProm1 cells when the tumor is already formed, we electroporated newborn mice with TP-Cherry and with plasmids expressing DTA under the control of Prom1-CreERT2-p27K^−^ (Fig. 4g). This enabled to control the DTA-mediated ablation of qProm1 cells by injection of Tamoxifen from P17 to P45 (Fig. 4g). The analysis of brain sections confirmed a reduction of qProm1 population (Fig. 4h, i), but the tumor size was surprisingly increased (Fig. 4j) upon tamoxifen injection. Interestingly, the ablation of qProm1 cells led to a reduction of infiltrating cells (Fig. 4k). Overall, our data suggest that qProm1 cells contribute to the infiltration/spread of tumor cells.

### Visualization of infiltrating quiescent cells in human pediatric brain cancer organoids

**Fig. 5:**
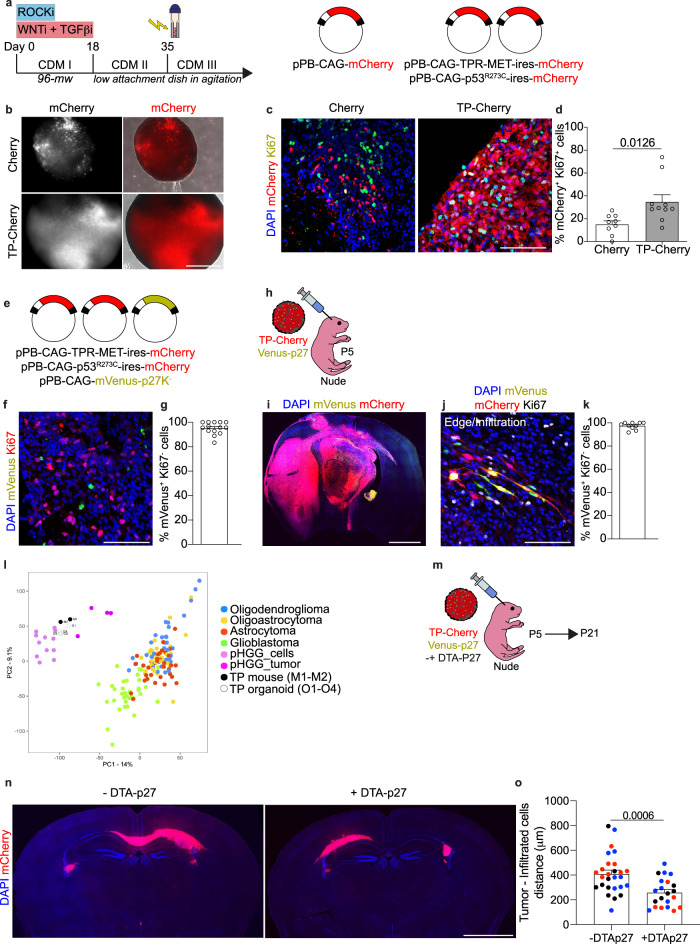
Direct visualization and targeting of infiltrating quiescent cells in human brain cancer organoids. **a** Scheme of the differentiation protocol of hiPSCs in forebrain organoids. At day 35 organoids were electroporated with pPB-CAG-mCherry (Cherry) or pPB-CAG-TPR-MET-ires-mCherry and pPB-CAG-p53^R273C^-ires-mCherry (TP-Cherry) and cultured for additional 30 days (30 dpe). **b** Images of electroporated organoids at 30 dpe (*n* = 7 organoids per type). **c**, **d** Images (**c**) and quantifications (**d**) of mCherry^+^ cells (red) co-expressing Ki67 (green) in organoids at 30 dpe electroporated with Cherry or TP-Cherry (*n* = 4 Cherry organoids, 368 cells; *n* = 6 TP-Cherry organoids, 1355 cells). Each dot represents an organoid section. **e** Electroporation of D35 organoids with pPB-CAG-TPR-MET-ires-mCherry, pPB-CAG-p53^R273C^-ires-mCherry and pPB-CAG-mVenus-p27K^−^ (TP-cherry/Venus-p27). Organoids were fixed at 30 dpe. **f**, **g** Images (**f**) and quantifications (**g**) of mVenus^+^ cells (green) not expressing Ki67 (red) in TP-Cherry/Venus-p27 organoids at 30 dpe (*n* = 8 organoids, 298 cells). **h** Grafting of TP-Cherry/Venus-p27 organoids in the brains of nude mice at postnatal day 5 (P5). Brains were dissected at humane endpoint. **i** Image of TP-Cherry/Venus-p27 organoid-derived brain tumor (*n* = 4 mice). **j**, **k** Image (**j**) and quantifications (**k**) of mCherry^+^ (red) and mVenus^+^ (green) cells not expressing Ki67 (gray) at infiltrating edge of organoid-derived tumors (*n* = 3 mice, 340 cells). **l** Principal Component Analysis of the transcriptomes of TP-induced tumors (M1 and M2) in mouse, TP-organoids grafts (O1, O2, O3, O4) and published human adult low- and high-grade glioma samples (oligodendroglioma, Oligoastrocytoma, astrocytoma and glioblastoma) and pediatric high-grade glioma samples. **m** Grafting of TP-Cherry/Venus-p27 organoids co-expressing or not DTA-p27 in the brains of nude mice. Brains were dissected at P21. **n** Images of TP-Cherry/Venus-p27 (without or with DTA-p27) organoid-derived brain tumors (*n* = 3 mice per condition). **o** Quantification of the distance of the infiltrated mCherry^+^ cells from the tumor edge in the brain sections of mice grafted with TP-Cherry/Venus-p27 without (*n* = 3 mice) or with DTA-p27 (*n* = 3 mice). The furthest three cells for each section were considered; each dot represents a cell and color code is applied to each mouse. Scale bars (**b**) 600 μm, (**c**, **f**, **j**) 100 μm, (**I**, **n**) 2 mm. Data are presented as mean ± SEM. P values were calculated by unpaired two-tailed Student’s *t* test (**d**, **o**). Source data are provided as a Source Data file.

Our data indicate that quiescent cells are involved in cancer invasiveness in mouse brain tumors, therefore we decided to validate our findings also in human tumors. To tackle this question, we developed a human brain cancer organoid model. TP-Cherry was electroporated in dorsal forebrain organoids differentiated from human induced pluripotent stem cells (hiPSCs) (Fig. 5a)^27^. Live imaging of 30 days-post electroporation (30 dpe) organoids showed a more diffuse mCherry^+^ fluorescence compared with the control (Fig. 5b). Histological characterization of the organoids confirmed that electroporation with TP-Cherry led to an increase in proliferating cells (Fig. 5c, d) as well as stem cell marker SOX2 (Supplementary Fig. 8a, b) and a reduction in neuronal marker NeuN (Supplementary Fig. 8c, d). To perform live imaging of the quiescent cells, we decided to drive Venus-p27expression under the control of a strong and ubiquitous promoter, such as CAG (Fig. 5e). We found that 89.2% of the quiescent mCherry^+^/mVenus^+^ cells co-expressed SOX2 (Supplementary Fig. 8e, f) and that 95.2% of mCherry^+^/mVenus^+^ cells were negative for proliferation marker Ki67 (Fig. 5f, g) confirming the visualization of slow-cycling/quiescent cells in brain cancer organoids. Since we used an ubiquitous promoter, we showed that mVenus labels mainly SOX2^+^ or S100B^+^ progenitor cells (Supplementary Fig. 8e–h) and a few NeuN^+^ neuronal cells (Supplementary Fig. 8i, j). We further characterized the infiltrative potential of those cells in vivo after grafting of TP-mCherry/mVenus-p27 electroporated organoids in the brain of nude mice (Fig. 5h–k, Supplementary Movie 2). In addition, we confirmed in grafted organoids that Venus labels mainly S100B^+^ progenitor cells (Supplementary Fig. 8k, l) rather than NeuN^+^ neuronal cells (Supplementary Fig. 8m, n). We then analyzed the methylation profile of DNA extracted from cancer organoids grafted in the brain of nude mice using the brain tumor classifier, a recently introduced tool that can complement the diagnostic process for brain cancer patients^28,29^. The three tested tumors clustered in the methylation class “*infantile hemispheric glioma”* with a calibrated score > 0.9 (Score 0.99, 0.97, 0.94 according to v12.3 brain tumor classifier). Furthermore, we also compared the transcriptome profiles of TP organoid-derived tumors and TP-induced mouse tumors (Fig. 2) with human adult and pediatric low- and high-grade brain cancers (Fig. 5l). Notably, both types of TP-induced tumors (organoid and mouse) clustered with pediatric high-grade glioma, with respect to other types of brain cancers (Fig. 5l). Afterwards, we performed live imaging of TP-Cherry/Venus-p27 co-cultured with non-electroporated brain organoids (Host) (Supplementary Fig. 8o, p), which revealed the infiltration of mCherry^+^/mVenus^+^ cells. We also confirmed the infiltration of mCherry^+^/mVenus^+^/Ki67^-^ cells in the cryosections of the co-cultured organoids (Supplementary Fig. 8q, r). Finally, we grafted TP-organoids co-electroporated with DTA-p27 to assay the effect of ablating quiescent cells in the infiltration of TP-Cherry-derived cells (Fig. 5m). The co-expression of DTA-p27 in TP-organoids led to a reduction in the infiltration of tumor cells (Fig. 5n, o). These data collectively establish an organoid-based model of pediatric brain cancer, suitable for investigating the role of quiescent infiltrating cells.

### Pharmacological targeting of quiescence cancer cells

**Fig. 6:**
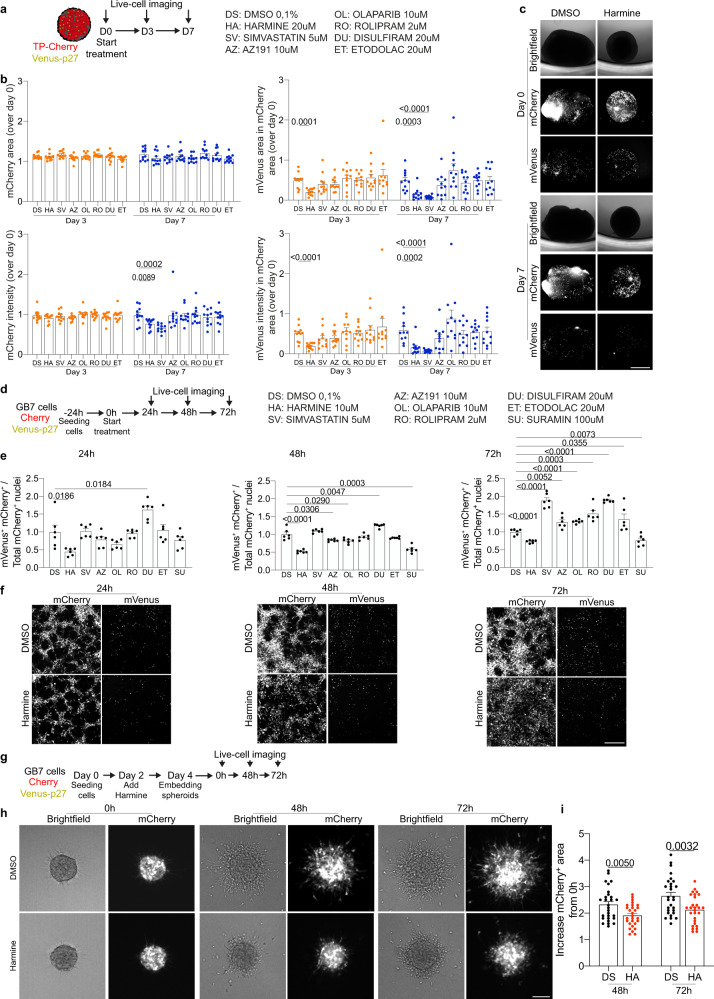
Pharmacological targeting of quiescent cells tested in human brain cancer organoids and GBM cell line reduces invasion. **a** TP-Cherry/Venus-p27 organoids at 30 dpe were treated with the listed drugs for 7 days. Organoids were live imaged at 0, 3 and 7 days of treatment. **b** Quantifications of mCherry^+^ and mVenus^+^ area and intensity at day 3 and day 7. Data were normalized to day 0. Each dot represents as single organoid (*n* = 11 organoids per condition). **c** Images of DMSO- or Harmine-treated organoids at day 0 and day 7 of treatment (*n* = 11 organoids per condition). **d** GB7 cells previously electroporated with pPB-CAG-mCherry (Cherry) and pPB-CAG-mVenus-p27K^−^ (Venus-p27) were seeded one day prior treatment. Cells were treated with the listed drugs for 72 h and imaged every 24 h. Nuclei were counterstained with Hoechst for counting the number of nuclei. For each drug 3 wells were imaged, and experiments were conducted in duplicate. **e** Quantification of mVenus^+^/mCherry^+^ cells over the total of mCherry^+^ cells 24 h, 48 h and 72 h post treatment. Each dot represents a well (*n* = 6 wells per condition). Data were normalized on the DMSO-treated cells on the same day. **f** Images of DMSO- or Harmine-treated cells at 24 h, 48 h and 72 h post treatment (*n* = *54* images per condition). **g** GB7 cell-derived spheroids were embedded in collagen type I and live imaged at 0 h, 48 h and 72 h post embedding. Treatment with DMSO or Harmine started 2 days before embedding (Day 2 during spheroid formation). **h**, **i** Images (**h**) and quantifications (**i**) of spheroid invasion in the collagen matrix. Invasion was measured as increased area of mCherry^+^ cells from 0 h. Each dot represents a spheroid (*n* = 28 spheroids per condition). Data were normalized on the same spheroid at 0 h timepoint. Scale bars (**c**) 1 mm, (**f**) 400 μm, (**h**) 100 μm. Data are presented as mean ± SEM. *P* values were calculated by unpaired two-tailed non-parametric Mann–Whitney test or unpaired two-tailed Student’s *t* test (**b, e, i**) depending on whether data were normally distributed. Source data are provided as a Source Data file.

The goal of chemotherapy treatments is to kill neoplastic cells, leading the cancer to slow down its growth. However, quiescent cells are poorly susceptible to classical antineoplastic drugs, due to their lower proliferation rate. Here, we sought to use the quiescence fluorescent sensor Venus-p27 to test the effect of selected drugs in ablating quiescent cells. We treated TP-mCherry (+ Venus-p27) organoids with two DYRK1A/B inhibitors (AZ191 and Harmine) and other drugs previously used to ablate cell quiescence or to inhibit EMT^30^ (Fig. 6a). Analysis of mCherry and mVenus positive area and intensity showed that Harmine and Simvastatin led to a reduction of mVenus signal compared with controls (Fig. 6b, c). We next tested the effects of the compounds on GB7 cancer stem cells (Fig. 6d, e and Supplementary Fig. 9a, b) and COMI cell lines (Supplementary Fig. 8c–g). To do so, we electroporated both lines with the same pPB plasmids described in the manuscript encoding for mCherry and Venus-p27 to allow imaging and analysis of the change in cell number and impact of the drugs on quiescent cells (Fig. 6d). We observed that Harmine consistently reduces quiescent cells when tested on both GB7 (Fig. 6e, f) and COMI (Supplementary Fig. 9c-g). We next sought to test whether the treatment with Harmine, along with reducing quiescent cells, affects the invasiveness of GBM lines cancer stem cells (Fig. 6g). Here, we show that Harmine treatment is indeed able to reduce the degree of invasion of GB7 spheroids (Fig. 6h, i). Overall, our data suggest that Harmine could be a therapeutic treatment for infiltrating high-grade gliomas.

## Discussion

Despite the presence of quiescent stem cells in human brain cancer has been already shown^5,7^ their role in cancer infiltration remains elusive. Here, we identified in human brain cancer samples a subpopulation of malignant cells expressing the stem marker Prominin-1 and characterized by a non-cycling transcriptome signature. Interestingly, the analysis of marker genes reveals that non-cycling Prom1^+^ cells express high levels of genes responsible for cell adhesion, filopodium assembly and cell spreading. For long time, Prominin-1 cells have been always considered as cancer stem cells with self-renewal capacity responsible for treatment resistance and cancer relapse^10–13^. Nevertheless, the observations obtained from the analysis of scRNA-seq data from patients led us to investigate the potential role of such cells in cancer invasion in vivo and in vitro models of brain cancer. To specifically study quiescent cells, we created a mouse model of brain cancer expressing TPR-MET and mutant p53 together with a well-known G0 sensor mVenus-p27K^−^. This allowed to directly visualize the quiescent Prom1^+^ or Sox2^+^ cells within the tumor and characterize their localization in the core or infiltrating edge tumor areas. We observed that quiescent cells located in the infiltrating edge adopted a mesenchymal-like/migratory morphology, with a strong accumulation of N-cadherin at the cell-cell contact. Despite previous observations linking quiescent tumor stem cells to the re-growth of the tumor after chemotherapy^4,8,9^, here we did not observe an increased proliferation in qProm1 progeny after TMZ treatment, maybe due to the time window we used. Interestingly, in our study TMZ treatment increased the percentage of qProm1-derived cells expressing OLIG2 in the core of the tumor. Since OLIG2 is also a key transcription factor required to reprogram differentiated glioma cells into highly tumorigenic glioma stem cells^26^, in the future it will be worth investigating whether anticancer drugs lead to a modification of the cell identity in human high-grade glioma. Furthermore, we specifically ablated quiescent Prom1^+^ cells by a cell cycle degradable DTA (DTA-p27K^−^). When the ablation was performed from the beginning of tumor growth, it led to increased mouse survival and to a reduction in tumor size as well as infiltration. On the other hand, ablation of qProm1^+^ cells after tumor establishment only affected the infiltration and spread of tumor cells. The system we conceived to specifically perform a CEll cycle-based Lineage Tracing and Ablation (CELTA) (Supplementary Fig. 10a, b) allowed us to reveal how qProm1^+^ cells contribute to the tumor infiltration (Supplementary Fig. 10c). We also created a human brain cancer organoid to specifically visualize and study the infiltration of quiescent cells. We showed that quiescent stem cells can be visualized in human brain cancer organoids and have infiltrative potential in vivo after injection in nude mice. Furthermore, we were able to live image the quiescent tumor cells invading a normal brain organoid in co-culture experiments. The impact of these tools lies into the possibility to build a platform for testing therapeutic strategies for tumor spread, easily exploitable also in other human and mouse cancer models. Here, we have exploited these models to identify drugs acting against quiescent cancer cells. Due to their lower rate of proliferation, quiescent cells are resistant to classical anticancer drugs. We identified Harmine as a potent inhibitor of quiescence cells state, also able to reduce the infiltration capacity of cancer cell. Harmine is an alkaloid originally isolated from the South American vine *Banisteriopsis caapi* and it is a putative inhibitor of ATP binding to the kinase pocket of DYRK1A^31^. Interestingly, DYRK1A complex is involved in the regulation of G0-G1 transition^32^ and Harmine has been shown to induce human beta cells to enter cell cycle^33,34^, suggesting a similar effect on quiescent cancer cells. Nevertheless, Harmine has also been shown to induce cell death and inhibition of proliferation in other brain cancer in vitro models^35,36^, therefore it cannot be excluded that Harmine might act on other targets. Here, we showed that Harmine could reduce the infiltrative potential of the quiescent cells in our system. Overall, our data suggest that Harmine may be a drug to be included in the therapeutic armamentarium against high-grade gliomas, capable of targeting slow-cycling/quiescent cells.

## Methods

### Analysis of single cell RNA-seq data

Single cell expression data were retrieved from Gene Expression Omnibus (Sample ID GSM3828672). Data analysis was performed with Seurat v4.0.4^37^, with default parameters. After Principal Component Analysis, cells were visualized with the UMAP dimension reduction technique. For each cell, the G1/S and G2/M expression scores were calculated with the “*AddModuleScore*” function of Seurat, based on signatures of G1/S and G2/M genes defined in^15^. Cell-cycle thresholds were defined first by fitting Gaussian distributions on empirical scores with the “*fitdistrplus”* R package, and then selecting the 45^th^ and 40^th^ percentile of the fitted distributions, respectively. Cells with both G1/S and G2/M expression scores below thresholds were considered as non-cycling. PROM1, SOX2 and OLIG2 positive and negative cells were defined based on the presence or absence of scRNA-seq reads, respectively. Marker genes of cell populations were identified with the “*FindMarkers*” function of Seurat, setting as thresholds: avg_log2FC > 0.5/<−0.5, pct.1 > 0.5, p_val_adj <0.05. Enrichment analysis of marker genes was performed with the “*clusterProfiler”* R package.

### Cloning

The hyperactive form of piggyBac transposase (pCMV-Hahy-pBase, pPBase) was donated by the Wellcome Sanger Institute. pPB-CAG-MCS-ires-mCherry plasmid was used as piggyBac donor backbone to clone by PCR other coding sequences. It was generated amplifying ires and mCherry coding sequence and then joining them by overlapping PCR. Ires-mCherry was then cloned into the piggyBac donor backbone pPB-CAG-MCS-ires-mVenus. TPR-MET was amplified by PCR from pBABE-puro-TPR-MET (Addgene, 10902). P53 (R273C) was cloned from pCMV-Neo-Bam-p53R273C (a gift from Alberto Inga). mVenus-p27K^−^ was amplified by PCR from pMXs-IP-mVenus-p27K^−^ (a gift from Toshihiko Oki and Toshio Kitamura). The human Sox2 (hSOX2) promoter was subcloned from pGL3-SOX2 plasmid (Addgene, 101761) into the piggyBac backbone replacing the CAG promoter (generating pPB-hSOX2). Mouse Prom1 (mProm1) promoter was amplified by PCR starting from mouse genomic DNA and cloned into the piggyBac donor vector (generating pPB-mProm1). The following primers were used to amplify the P2 element of mouse Prominin1 promoter, as previously described:^14^

Fw: TTCTTTGATATCGGTACCGGTCCAATCAGTGCGCTCAGAC;

Rev: TTCTTTCTCGAGAAGCTTCCTCTCCGGTCCAGCTCTCCT.

CreERT2-p27k^−^ was generated amplifying CreERT2 (from pPB-hSynI-CreER-ires-Venus) and p27K^−^ (from pPB-CAG-mVenus-p27K^−^) and then joining them by overlapping PCR. CreERT2-p27K^−^ was then cloned into the piggyBac donor backbone pPB-CAG. mVenus-p27K^−^ or CreERT2-p27K^−^ were cloned from their respective pPB-CAG into pPB-hSOX2 or pPB-mProm1. DTA-p27K^−^ was generated amplifying DTA (from pDTA-TK, Addgene 22677) and p27K^−^ (from pPB-CAG-mVenus-p27K^−^) and then joining them by overlapping PCR. DTA-p27K^−^ was then cloned into the piggyBac donor backbone pPB-CAG, pPB-mProm1 or pPB-hSOX2. The primer sequences used to generate the fusion proteins are listed below:

CreERT2-p27K^−^

CreERT2: Fw: 5′-TTCTTTGCTAGCGCCACCATGTCCAATTTACTGACCGTACA-3′;

Rev: 5′-CGCCAGTGTGATGGATATCCAGCTGTGGCAGGGAAACC-3′

p27K^−^ Fw: 5′-GGTTTCCCTGCCACAGCTGGATATCCATCACACTGGCG-3′

Rev: 5′-TTCTTTGAATTCTCATTACGTCTGGCGTCGAA-3′

DTA-p27K ^-^

DTA Fw: 5′-TTTCTTGCTAGCGCCACCATGGATGATGTTGTTGATTCTTCTAAATC-3′

Rev: 5′-CGCCAGTGTGATGGATATCCAGATCGCCTGACACGATTTCC-3′

p27K^−^ Fw: 5’-GAAATCGTGTCAGGCGATCTGGATATCCATCACACTGGCG-3′

Rev: 5′-TTCTTT*GAATTC*TCATTACGTCTGGCGTCGAA-3′

pPB-CAG-LSL-MCS plasmid, used as donor backbone to clone other coding sequences, was generated inserting a loxP-STOP-loxP (LSL) cassette between CAG promoter and Multiple Cloning Sites. DTA and mVenus were cloned by PCR generating pPB-CAG-LSL-DTA and pPB-CAG-LSL-mVenus, respectively. All constructs were verified by DNA sequencing.

### Mice husbandry

Mice were housed in a certified Animal Facility in accordance with European Guidelines. Mice were housed at 12 h/12 h Dark/Light cycle, 22 °C temperature. The study was carried out in accordance with the recommendations of the Interna Review Board of University of Trento and European Guidelines. The experiments were approved by the Italian Ministry of Health as conforming to the relevant regulatory standards. CD1 and CD1-Nude mice were purchased from Charles River Laboratories. Prom1-CreERT2 mice (JAX# 017743) were purchased from The Jackson Laboratory. Temozolomide (Sigma-Aldrich, T2577) was intraperitoneally injected at 82.5 mg/kg/day for five days. Tamoxifen (Alfa Aesar, J63509) was intraperitoneally injected at 50 mg/kg/day. EdU (Life technologies, A10044) was intraperitoneally injected at 50 mg/kg. Animals were sacrificed at humane endpoint as they displayed signs of morbidity (loss of weight, loss of coordination, hunched posture, ruffled fur) or otherwise stated in the text or figures. The maximal tumor size/burden was not exceeded.

### In vivo electroporation

Postnatal day 2 (P2) CD1 mice were anesthetized on ice for 2 min, placed on a stage in a stereotactic apparatus and injected at the following coordinates (from lambda): −1,5 D/V, + 0.8 M/L, + 1.5 A/P. The DNA mix was prepared at a concentration of 5 µg/µl, with the pPBase and the piggyBac donor plasmids mixed at 1:4 ratio. 2 µl of DNA mix were injected using a pulled glass capillary and a FemtoJet microinjector (Eppendorf). DNA electroporation was performed with tweezers electrodes using the following parameters: 100 V, 50 ms/pulse, 1000 ms intervals, 5 electric squared pulses.

### Tissue preparation and immunofluorescence

Mice were perfused through intraventricular injection of 4% paraformaldehyde (PFA), brains were dissected and post-fixed for the following 24 h. Mouse brains were subsequently washed in PBS 1X, embedded in 5% agarose, and sectioned using Leica VT 1200 vibratome at a thickness of 60 µm. Brain sections were then washed with 0.3% Triton X-100 (Sigma) in PBS 1X and permeabilized in Sodium dodecyl sulfate 1X for 15 min. Primary antibodies were diluted in an antibody solution consisting of 0.3% Triton X-100 and 3% Goat Serum (Sigma, G6767) in PBS 1X, and incubated overnight. Secondary antibodies were incubated for 90 min at room temperature, diluted in the same antibody solution, and nuclei were counterstained with 1 µg/ml DAPI (Sigma).

EdU detection was performed after immunostaining. Briefly, following incubation with the secondary antibody, brain sections were post-fixed with 4% PFA for 15 min at room temperature, washed with 3% bovine serum albumin (BSA) (Seqens IVD, 1000-70) in PBS 1X for 10 min and permeabilized with 0.5% Triton X-100 for 20 min. Sections were then incubated for 30 min with a reaction mix containing PBS 1X, 4 mM CuSO4, Alexa Fluor^®^ 647 Azide (Life Technologies, A10277) and 100 mM sodium ascorbate (Sigma). Sections were washed and nuclei were counterstained with 1 µg/ml DAPI. The antibodies used for immunofluorescence are listed below:

**Table:** 

Antibodies	Host	Dilution	Company	Cat. N.
GFP	Chicken	1:1500	Abcam	Ab13970
Ki67	Rabbit	1:500	Abcam	Ab15580
Ki67	Rat	1:500	Thermofisher Scientific	14-5698-82
Sox2	Rabbit	1:500	Abcam	Ab97959
Olig2	Rabbit	1:1500	Sigma Aldrich	AB9610
NeuN	Mouse	1:500	Sigma Aldrich	MAB377
N-cadherin	Rabbit	1:1000	Abcam	Ab18203
S100β	Rabbit	1:500	Abcam	Ab52642
Alexa Fluor 405 goat anti-rabbit IgG	Goat	1:500	Thermofisher Scientific	A31556
Alexa Fluor 488 goat anti-chicken IgY	Goat	1:500	Thermofisher Scientific	A11039
Alexa Fluor 546 goat anti-mouse IgG	Goat	1:500	Thermofisher Scientific	A11030
Alexa Fluor 546 goat anti-rabbit IgG	Goat	1:500	Thermofisher Scientific	A11035
Alexa Fluor 647 goat anti-mouse IgG	Goat	1:500	Thermofisher Scientific	A21235
Alexa Fluor 647 goat anti-rabbit IgG	Goat	1:500	Thermofisher Scientific	A21245
Alexa Fluor 647 goat anti-rat IgG	Goat	1:500	Thermofisher Scientific	A21247

### RNA extraction and RNA sequencing and analysis

Fresh brains were dissected, and tumor tissues were carefully isolated by a fluorescent binocular microscope, snap frozen in liquid nitrogen and stored at −80 °C. Total RNA was isolated from tissues with TRIzol Reagent (Invitrogen, 15596018) according to the manufacturer’s instructions. Then, RNA quality was controlled with the High Sensitivity RNA Assay at the 2100 Bioanalyzer (Agilent, G2939BA) and the extracted RNA was stored at −80 °C until the RNA-seq analysis. Sequencing was performed on the Novaseq 6000 instrument (Illumina, San Diego, CA) on an SP flowcell, producing 900 M single reads 100nt. Sequencing reads from the FASTQ files were then aligned onto the appropriate reference genome (mouse GRCm38/human GRCh38). Instead, the FASTQ files of the 16 pediatric samples were downloaded from the EGA archive (https://ega-archive.org/datasets/EGAD00001004116) and those of the 140 adult samples (35 astrocytoma, 35 glioblastoma, 35 oligoastrocytoma and 35 oligodendroglioma) were obtained from The Cancer Genome Atlas (TCGA). To obtain the PCA in Fig. 5l, the aligner STAR (PMID:23104886) version 2.7.7a was used, setting the parameters outFilterScoreMinOverLread and outFilterMatchNminOVerLread to the value 0.33, the resulting SAM files were sorted and converted to BAM files using SAMTools (PMID:19505943) and transcripts counts were computed using the “*featureCounts”* function available from the Rsubread R package (PMID: 30783653). Instead, for the other PCA of Figure S4A the alignment of sequencing reads was performed with the transcript abundance quantifier Salmon (PMID: 28263959) version 1.4.0 and the R package tximeta (PMID: 32097405) was used to execute counts summarization to the gene level. In both cases then, transcripts with a raw count lower than 20 in all biological replicates across all considered conditions were excluded. TMM (Trimmed Mean of M values) normalization and CPM (Counts Per Million) conversion were performed with the edgeR R package (PMID:19910308) in order to obtain normalized transcript levels.

### Brain organoids culture, electroporation and grafting

Human induced pluripotent stem cells (hiPSCs, a gift from Domenico Delia) were maintained in self renewal on a layer of Geltrex (Gibco, A14133-01), in Essential 8 Basal Medium (Gibco, A15169-01) supplemented with E8 Supplement (Gibco, A15171-01) and P/S (Penicillin 100 Units/ml, Streptomycin 100 μg/ml, GIBCO, 15140-122). All cells were mycoplasma free. hiPSCs were dissociated with EDTA (Invitrogen, 15575-038) 0.5 mM, pH 8.0, to maintain cell clusters. Dorsal forebrain organoids were generated modifying a previously described protocol for dorsal forebrain differentiation^27^. Specifically, cerebral organoids were cultured in non-tissue-treated 100 mm or 60 mm dishes (Sarstedt) in cortical differentiation medium (either CDM3 or CDM4, based on protocol guidelines)^27^ supplemented with 1% Matrigel from day 35 onwards. On day 35, organoids were electroporated with different combinations of plasmids as previously described^38^. In particular, 15-20 organoids per condition were electroporated with 100 µg of DNA mix containing the piggyBac transposase and the donor plasmids mixed at a 1:4 ratio diluted in 100 µl of Buffer 5^38^. For co-culture experiment, one organoid at 30 days post electroporation (30 dpe) was culture in close contact with one non-electroporated organoid in a µ-Plate 96-well black (Ibidi, 89621). The co-cultures were monitored and acquired every day for 1 month with daily changing medium. Organoids were fixed overnight with 4% PFA at 30 dpe or 60 dpe (for co-culture experiment), then cryopreserved with 30% sucrose overnight and embedded in Frozen Section Compound (Leica, 3801480). Organoids were sectioned at 20 µm using Thermo Scientific HM525 NX cryostat. Immunofluorescence was performed as previously described for mouse sections. For grafting experiments, three 30 dpe organoids were mechanically dissociated in 200 µl of CDM3 and 8 µl of solution was injected with a 26s-gauge Hamilton Syringe (80300/00). Postnatal day 5 (P5) Nude mice were anesthetized on ice for 2 min, placed on a stage in a stereotactic apparatus and injected at the following coordinates (from lambda): −1,5 D/V, + 1.2 M/L, + 1.5 A/P. Animals were sacrificed at humane endpoint.

### GBM cell lines and nucleofection

Human glioblastoma stem cell line GB7 (from Luciano Conti)^39^ were cultured on vitronectin-coated dishes in Euromed-N media (Euroclone), supplemented with GlutaMAX (1%; Thermo Fisher Scientific, 35050038), B27 supplement (2%; Thermo Fisher Scientific, 17504044), N2 (1%; Thermo Fisher Scientific, 17502001), P/S (Penicillin 100 Units/ml, Streptomycin 100 μg/ml, GIBCO, 15140-122), recombinant human fibroblast growth factor-2 (bFGF) (20 ng/mL, PeproTech, 100-18B), and recombinant human epidermal growth factor (EGF) (20 ng/mL; PeproTech, AF-100-15). All cells were mycoplasma free.

Human glioblastoma stem cell line COMI (gift from Antonio Daga, Azienda Ospedaliera Universitaria San Martino di Genova, Italy), were cultured on laminin-coated dishes in DMEM/F-12 and Neurobasal media (Thermo Fisher Scientific, 1:1 ratio), supplemented with GlutaMAX (1%; Thermo Fisher Scientific, 35050038), B27 supplement (1%; Thermo Fisher Scientific, 17504044), P/S (Penicillin 100 Units/ml, Streptomycin 100 μg/ml, GIBCO, 15140-122), recombinant human fibroblast growth factor-2 (bFGF) (10 ng/mL; PeproTech, 100-18B), recombinant human epidermal growth factor (EGF) (20 ng/mL; PeproTech, AF-100-15) and heparin (2 mg/mL; Sigma Aldrich, H3149). All cells were mycoplasma free. For nucleofection, in total 1 × 10^6^ cells were nucleofected with 5 µg of plasmid DNA in 100 µl of nucleofection buffer using the A-033 (High-efficiency) program and a Nucleofector 2b device (Amaxa). Cells were expanded for further experiments.

### Drug treatments

Organoids electroporated with TP-Cherry, CAG-Venus-p27 were transferred in a non-treated 48-well plate (VWR, 734-2780) at 30 dpe and treated for 7 days, with changing medium every 48 h. The following drugs were tested, using DMSO as a control: AZ191 (HY-12277), Harmine (HY-N0737A), Simvastatin (HY-17502), Olaparib (HY-10162), Rolipram (HY-16900), Disulfiram (HY-B0240), Etodolac (HY-76251). The concentrations used in the experiments are stated in Fig. 6a.

Organoid images were acquired at day 0, day 3 and day 7 (taken Day 0 as the day in which the treatment started) using ImageXpress Micro Confocal High-Content Imaging System (Molecular Devices): a z-series composed by 70 z-steps with 10 µm step size, using 4x Plan Apo Objective in confocal mode for two fluorescent channels (mVenus and mCherry). To obtain the whole organoid images, the object XY centroids were automatically determined by QuickID procedure. In brief, a low magnification image of the entire well was acquired using 2x Plan Apo Objective in Widefield mode in the red fluorescent channel. The objects were identified by automated segmentation and the XY centroids positions were extracted. These parameters were used to center the objects in the final confocal acquisition. The final organoids images were analyzed using MetaXpress® High-Content Image Acquisition and Analysis Software (Molecular Devices). For each timepoint (day 0, 3, 7) and each reporter (mVenus, mCherry), the organoid fluorescence integrated intensity (sum of all fluorescence intensity of each pixel) and total area were measured starting from the maximum intensity projection of the acquired z-series. For each parameter, the reported value of every single organoid at Day 3 and Day 7 was normalized over its own value at Day 0.

GB7 or COMI cells were respectively seeded at the density of 2 × 10^4^ or 8 × 10^3^ cells per well one day prior starting the treatment in 96-well plates considering 3 wells per conditions and one plate for each timepoint. On day 0, medium supplemented with the aforementioned drugs (Fig. 6d) was replaced to each well and changed every 48 h. 1 h prior imaging cells were counterstained with Hoechst. Images were acquired using ImageXpress Micro Confocal High-Content Imaging System (Molecular Devices): one stack confocal image using 10x Plan Apo Objective in confocal mode for three fluorescent channels (Hoechst, mVenus and mCherry) at 24 h, 48 h and 72 h. For each condition 3 wells were imaged, and 9 images were taken for each well. The final images were analyzed using MetaXpress® High-Content Image Acquisition and Analysis Software (Molecular Devices). For each timepoint, Hoechst was used to count the total nuclei and among them the mCherry^+^/mVenus^+^ and mCherry^+^/mVenus^-^ were calculated. For each timepoint cell numbers were normalized to DMSO-treated cells on the same day.

### Invasion assay

GB7 were cultured in low-attachment 96-well plate (VWR 734-2781) (2,5 × 10^4^ cells per well) in DMEM/F12 (Thermo Fisher Scientific), supplemented with GlutaMAX (1%; Thermo Fisher Scientific, 35050038), B27 supplement (2%; Thermo Fisher Scientific, 17504044), N2 (1%; Thermo Fisher Scientific, 17502001), P/S (Penicillin 100 Units/ml, Streptomycin 100 μg/ml, GIBCO, 15140-122), recombinant human fibroblast growth factor-2 (bFGF) (20 ng/mL, PeproTech, 100-18B), and recombinant human epidermal growth factor (EGF) (20 ng/mL; PeproTech, AF-100-15). After 4 days, spheroids were embedded in 1,5 mg/ml bovine collagen type I (Advanced BioMatrix, 5005) and imaged from day 0 using Nikon Eclipse Ti2 equipped with CREST Optics X-Light V2 Spinning (31 z-steps with 2,5 µm step size). Invasion progression was calculated as increased area of mCherry over day 0 using a custom-made macro for ImageJ software (v.1.53i).

### Genomic DNA extraction and methylation analysis

Grafted brains were freshly dissected, tumors were separated from brain tissue by a fluorescent binocular microscope. Tumor biopsies were snap frozen in liquid nitrogen and stored at −80 °C. Tumor biopsies were lysed in lysis buffer (20 mM EDTA, 10 mM Tris, 200 mM NaCl, 0.2% Triton X-100, 100 µg/ml Proteinase K, pH 8.0) for 2 h at 37 °C. Genomic DNA was extracted with phenol-chloroform and precipitated with isopropanol. DNA methylation profiling was performed as previously described^38^.

### Imaging

Brain and organoid sections were imaged on either Leica TCS Sp8 or Nikon Eclipse Ti2 equipped with CREST Optics X-Light V2 Spinning Disk. Co-cultured organoids were imaged on Nikon Eclipse Ti2 equipped with CREST Optics X-Light V2 Spinning Disk. Imaging data were collected using Leica Application Suite X (LAS X) on Leica TCS Sp8 confocal microscope or NIS-Elemelton on CREST Optics X-Light V2 Spinning Disk. Images are presented as maximum intensity projection images or a single Z stack. Images were processed using ImageJ software.

### Quantification of area and infiltrated cells in mouse brain sections

For the quantification of mVenus^+^, Cherry^+^ and DAPI^+^ area, whole brain sections were acquired with Nikon Eclipse Ti2 equipped with CREST Optics X-Light V2 Spinning Disk generating mosaic images. A defined threshold was applied to each separated channel to create binary images. Measurement of the area was limited to the threshold. At least 3 sections per brain were analyzed (numbers of mice analyzed are stated in the relative figure legends). For the quantification of mVenus^+^ or Cherry^+^ infiltrated cells, the distance between infiltrated cells and tumor edge was measured for at least 6 cells and the furthest 3 cells for each section were considered.

For the quantification of Sox2 + /Venus+ cells and NeuN + /Venus+ cells, brain sections were acquired using Leica TCS Sp8. For each section, up to two z-stacks were quantified separately. The area of mCherry+ cells (reported in mm^2^) was measured applying a defined threshold to create binary images. The number of double positive (Sox2 + /Venus + /Ki67−, NeuN + /Venus + /Ki67−) or triple positive (Sox2 + /Venus + /Ki67 + , NeuN + /Venus + /Ki67+) cells for each z-stack was normalized over the area of mCherry+ cells calculated for the same z-stack. All the image measurements were performed with ImageJ software.

### Statistical analysis

All statistical analyses were performed with GraphPad Prism 9 software. All in vivo experiments were performed using independent biological replicates, as indicated in the text and figures for each experiment. All in vitro experiments were performed at least twice using independent biological replicates or additional cell lines. All the experiments showed similar results.

Quantitative data were presented as mean ± SEM. with median and quartiles as indicated in figure legends. Prior to statistical significance testing, data were tested for normal distribution. For normally distributed data, the unpaired Student’s test was used for 2 groups. For data not following a normal distribution, unpaired two-tailed non-parametric Mann–Whitney test was used for 2 groups and the Kruskal–Wallis test followed by Dunn’s test for more than 2 groups. Kaplan-Meier survival curve followed by Log-rank (Mantel-Cox) test was used for testing difference in the survival of mice.

## Supplementary information

Supplementary Information

Description of Additional Supplementary Files

Supplementary Movie 1

Supplementary Movie 2

Supplementary Data 1

Supplementary Data 2

## Data Availability

All data associated with this study are in the article and/or supplementary information. RNA-seq data generated in this study have been deposited at 10.5281/zenodo.6794170. Additionally, the following public databases were used: Single cell RNA-seq from adult and pediatric GBM samples were obtained from GEO (GSE131928); bulk RNA-seq from 16 pediatric samples were downloaded from the EGA archive (https://ega-archive.org/datasets/EGAD00001004116); bulk RNA-seq from 140 adult samples (35 astrocytoma, 35 glioblastoma, 35 oligoastrocytoma and 35 oligodendroglioma) were obtained from The Cancer Genome Atlas (TCGA); bulk RNA-seq from mice tumor samples were obtained from GEO (GSE151414). Remaining data are available within the article, supplementary information, or source data file. All the plasmids developed in this study could be made available upon request to luca.tiberi@unint.it. Source data are provided in this article. Source data are provided with this paper.

## Source data

Source Data
