# Supplementary material for: A slow-cycling/quiescent cells subpopulation is involved in glioma invasiveness
Source: Nat Commun. 2022 Aug 15;13:4767. doi: 10.1038/s41467-022-32448-0 (PMC9378633; doi:10.1038/s41467-022-32448-0)
Supplement: Supplementary file 1 — Supplementary Information [file 41467_2022_32448_MOESM1_ESM.pdf]

Supplementary Information

A slow-cycling/quiescent cells subpopulation is involved in glioma invasiveness

Francesco Antonica*, Lucia Santomaso, Davide Pernici, Linda Petrucci, Giuseppe Aiello, Alessandro Cutarelli, Luciano Conti, Alessandro Romanel, Evelina Miele, Toma Tebaldi and Luca Tiberi*.

*Correspondence: francesco.antonica@unitn.it, luca.tiberi@unitn.it

This file includes:

Supplementary Figures S1-S10

Supplementary Figure 1

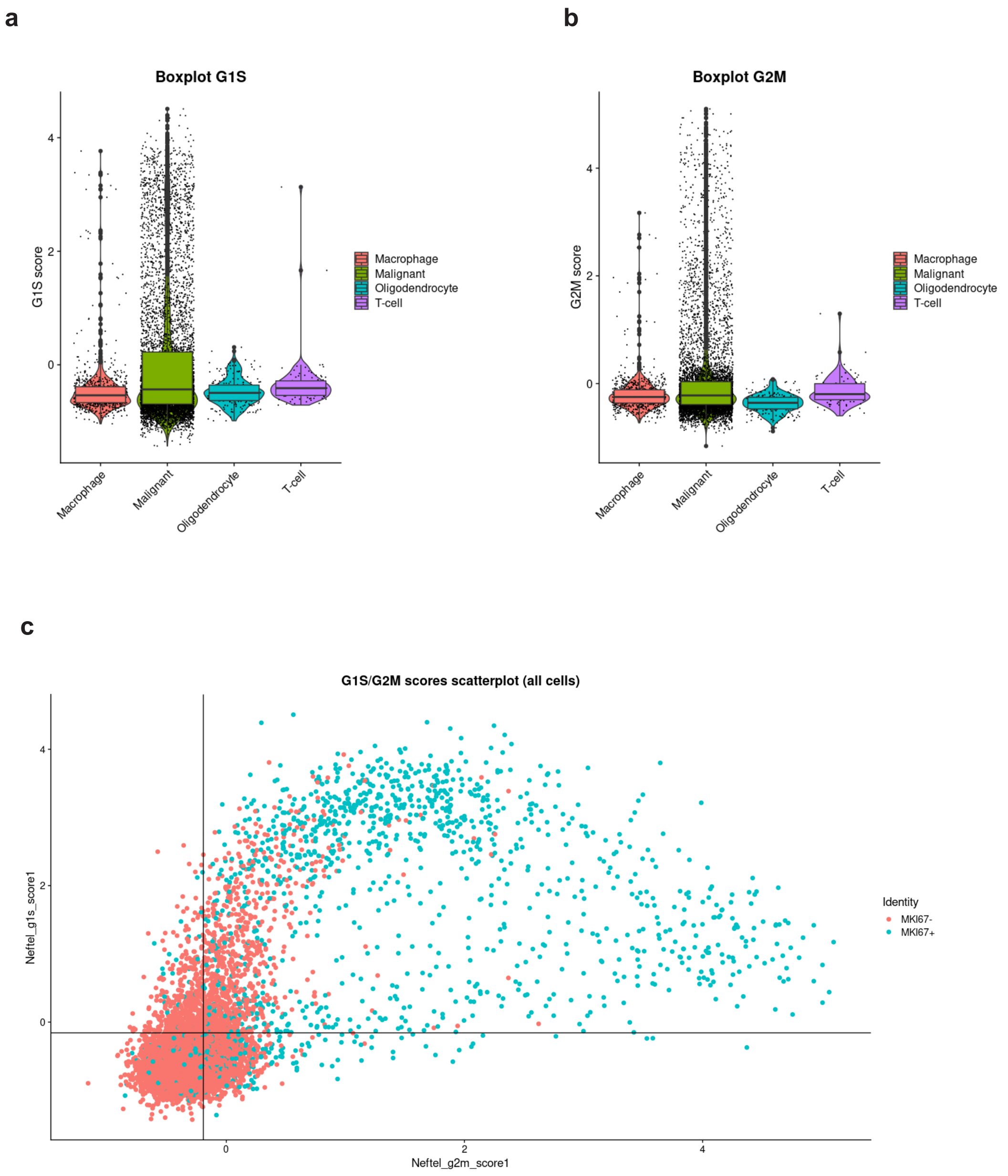

Supplementary Figure 1. Related to Figure 1. Definition of cycling and non-cycling cells in glioblastoma single cell data. (**a**) Distributions of G1/S expression scores in glioblastoma cell populations. (**b**) Distributions of G2/M expression scores in glioblastoma cell populations. (*n* = 754 macrophages; *n* = 6863 malignant cells; *n* = 219 oligodendrocytes; *n* = 94 T-cells). Boxplot generated with the function ggplot2::geom_boxplot (https://ggplot2.tidyverse.org/reference/geom_boxplot.html). Lower whisker = smallest observation greater than or equal to lower hinge - 1.5 * IQR; lower hinge = 25% quantile; middle bar = median, 50% quantile; upper hinge = 75% quantile; upper whisker = largest observation less than or equal to upper hinge + 1.5 * IQR. (**c**) Scatterplot of glioblastoma cells based on their G1/S and G2/M expression scores (Y and X, respectively). Score thresholds used to define cycling vs non-cycling cells are plotted. Cells are color-coded according to the expression of the proliferation marker Ki67.

Supplementary Figure 2

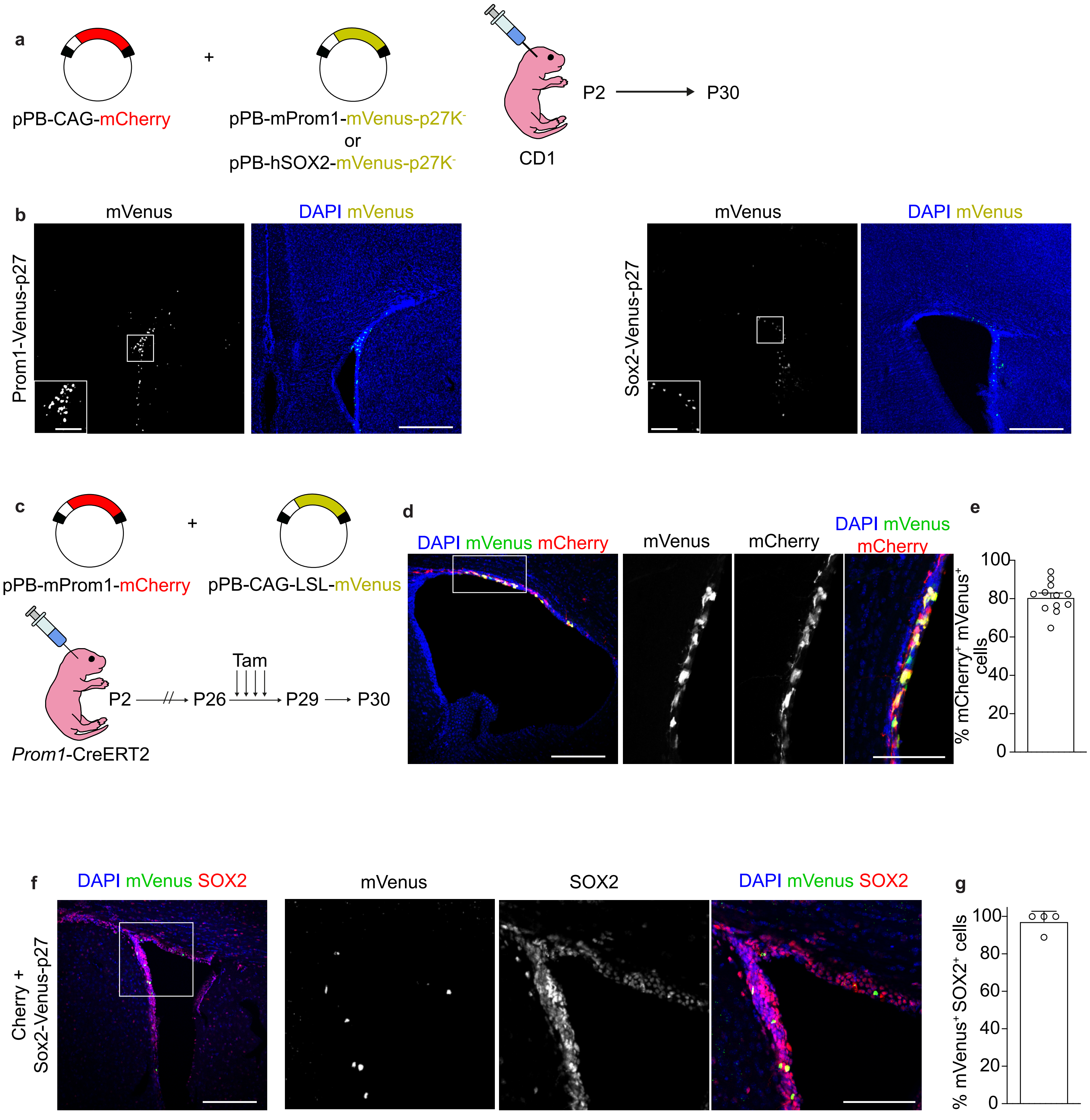

Supplementary Figure 2. Related to Figure 2. Test of the specificity of *mProm1* and *hSOX2* promoters in mouse brain. (**a**) Electroporation of cells in the SVZ of P2 CD1 mice with pPB-CAG-mCherry and pPB-mProm1-mVenus-p27K$^-$ (Prom1-Venus-p27) or pPB-hSOX2-mVenus-p27K$^-$ (Sox2-Venus-p27). Brains were dissected at P30. (**b**) Images of the expression of the reporter mVenus-p27K$^-$ under the control of Prom1 (*n* = 4 mice) or Sox2 promoter (*n* = 4 mice). (**c**) Electroporation of cells in the SVZ of P2 *Prom1*-CreERT2 mice with pPB-mProm1-mCherry and pPB-CAG-LSL-mVenus. Mice were injected daily with Tamoxifen from P26 to P29 and brains were dissected at P30. (**d, e**) Images (**d**) and quantifications (**e**) of mCherry$^+$ cells expressing mVenus (*n* = 3 mice, 213 cells). (**f, g**) Images (**f**) and quantifications (**g**) of mVenus$^+$ cells expressing SOX2 (*n* = 2 mice, 36 cells). Scale bars (**b**) 200 μm and 100 μm (ROI), (d) 200 μm and 100 μm (ROI), (**f**) 200 μm and 100 μm (ROI). Data are presented as mean ± SEM. Source data are provided as a Source Data file.

Supplementary Figure 3

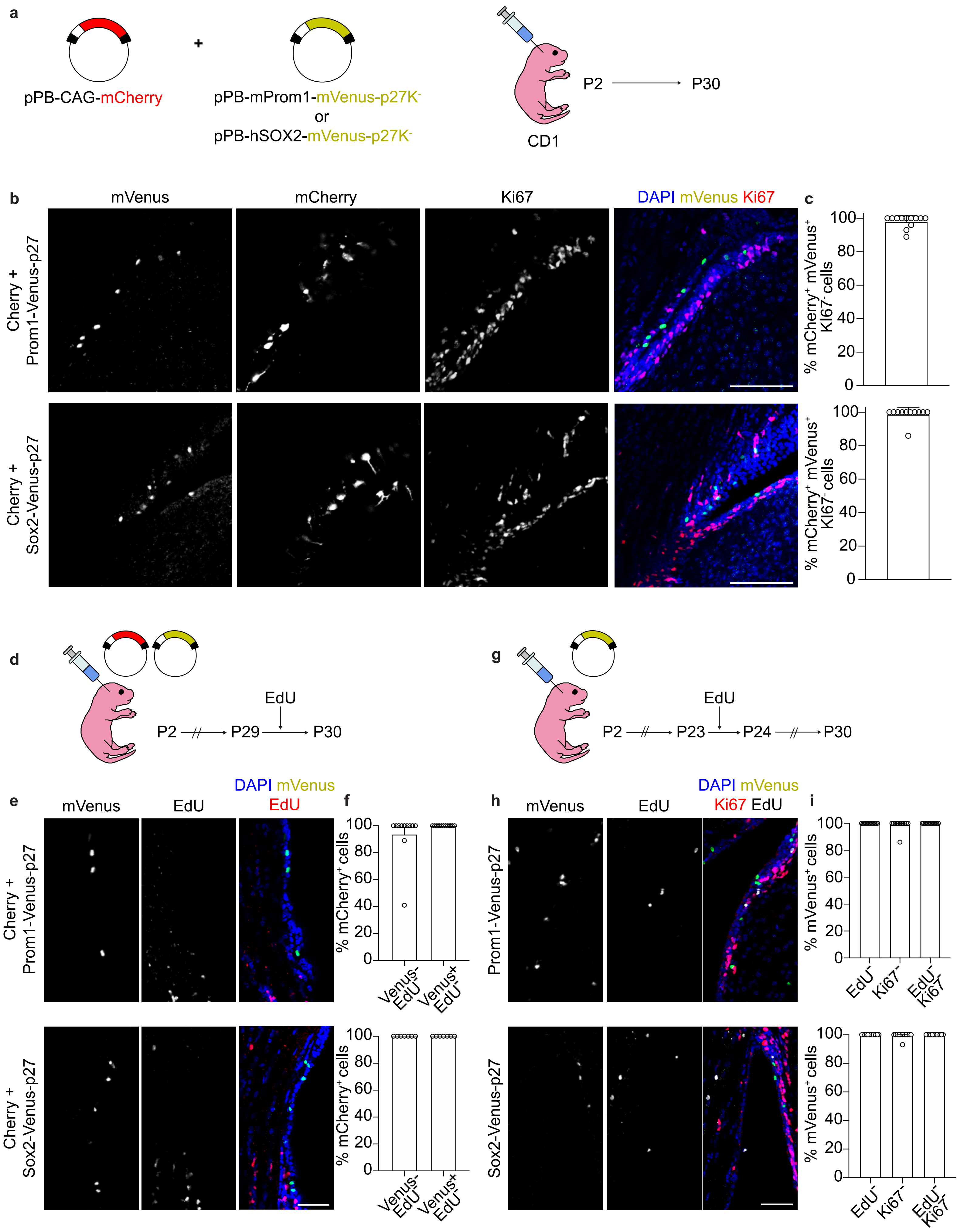

Supplementary Figure 3. Related to Figure 2. Test of the cell cycle specificity of mVenus-p27K$^-$ reporter in mouse brain. (**a**) Electroporation of cells in the SVZ of P2 CD1 mice with pPB-CAG-mCherry and pPB-mProm1-mVenus-p27K$^-$ or pPB-hSOX2-mVenus-p27K$^-$. Brains were dissected at P30. (**b, c**) Images (**b**) and quantifications (**c**) of mVenus$^+$ cells (green) not expressing Ki67 (red) in the SVZ of mice expressing Prom1-Venus-p27 (*n* = 3 mice, 171 cells) or Sox2-Venus-p27 (*n* = 3 mice, 84 cells). (**d**) Electroporation of cells in the SVZ of P2 CD1 mice with pPB-CAG-mCherry and pPB-mProm1-mVenus-p27K$^-$ or pPB-hSOX2-mVenus-p27K$^-$. Mice were injected with EdU at P29 and brains were dissected at P30. (**e, f**) Images (**e**) and quantifications (**f**) of mCherry$^+$ cells not labelled with EdU (red) and expressing or not mVenus (green) in the SVZ of mice expressing Prom1-Venus-p27 (*n* = 4 mice, 104 cells) or Sox2-Venus-p27 (*n* = 2 mice, 46 cells). (**g**) Electroporation of cells in the SVZ of P2 CD1 mice with pPB-mProm1-mVenus-p27K$^-$ or pPB-hSOX2-mVenus-p27K$^-$. Mice were injected with EdU at P23 and brains were dissected at P30. (**h, i**) Images (**h**) and quantifications (**i**) of mVenus$^+$ cells (green) not labelled with EdU (gray) and/or not expressing Ki67 (red) in the SVZ of mice expressing Prom1-Venus-p27 (*n* = 3 mice, 120 cells) or Sox2-Venus-p27 (*n* = 3 mice, 97 cells). Scale bars (**b**) 100 μm, (**e, h**) 50 μm. Data are presented as mean ± SEM. Source data are provided as a Source Data file.

Supplementary Figure 4

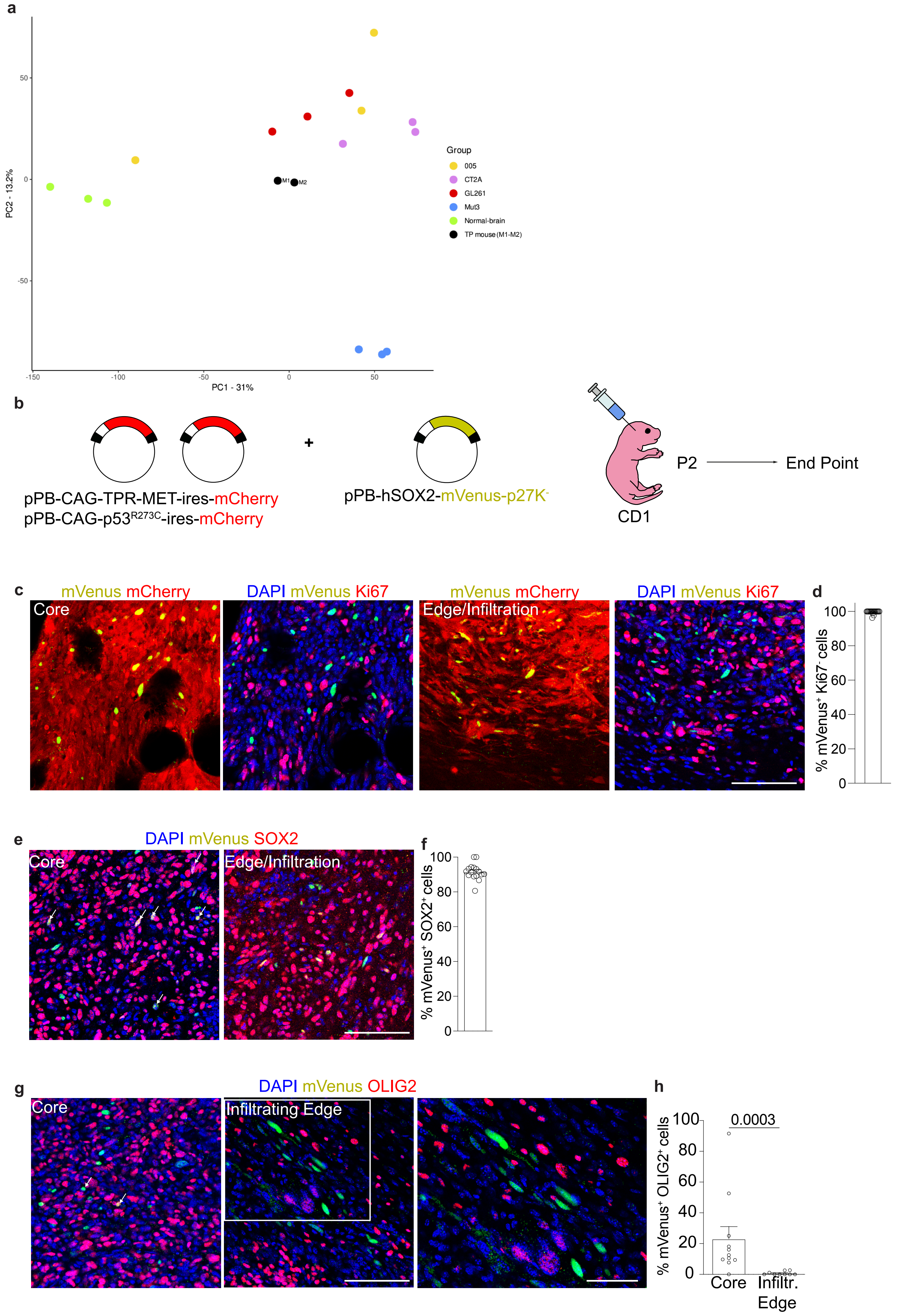

Supplementary Figure 4. Related to Figure 2. Characterization of TP-induced tumors. (**a**) RNA-seq analysis of TP-induced tumors (M1 and M2) and published datasets of mouse glioma and normal brain[23]. Principal Component Analysis (PCA) of TP-induced tumors (TP), published datasets of RNA-seq of mouse glioma (CT2A, GL261, Mut3, 005) and normal brain. (**b**) Electroporation of cells in the SVZ of P2 CD1 mice with pPB-CAG-TPR-MET-ires-mCherry and pPB-CAG-$p53^{R273C}$-ires-mCherry (TP-Cherry) together with pPB-hSOX2-mVenus-$p27K^{-}$ (Sox2-Venus-p27). Mice were sacrificed at humane endpoint. (**c, d**) Images (**c**) and quantifications (**d**) of $mVenus^{+}$ cells (green) not expressing Ki67 (red) in the core and edge/infiltrative regions of the $mCherry^{+}$ tumors expressing Sox2-Venus-p27 (n = 3 mice, 1217 cells). (**e, f**) Images (**e**) and quantifications (**f**) of $mVenus^{+}$ cells (green) expressing SOX2 (red) in the core and edge/infiltrative regions of the $mCherry^{+}$ tumors expressing Sox2-Venus-p27 (n = 3 mice, 627 cells). (**g, h**) Images (**g**) and quantifications (**h**) of $mVenus^{+}$ cells (green) expressing OLIG2 (red) in the core and edge/infiltrative regions of the $mCherry^{+}$ tumors expressing Sox2-Venus-p27 (for core n = 3 mice, 907 cells; for infiltrating edge n = 3 mice, 610 cells). Scale bars (**c, e, g**) 100 μm, (**g**, ROI) 50 μm. Data are presented as mean $\pm$ SEM. P values were calculated by unpaired two-tailed non-parametric Mann-Whitney test (**h**). Source data are provided as a Source Data file.

Supplementary Figure 5

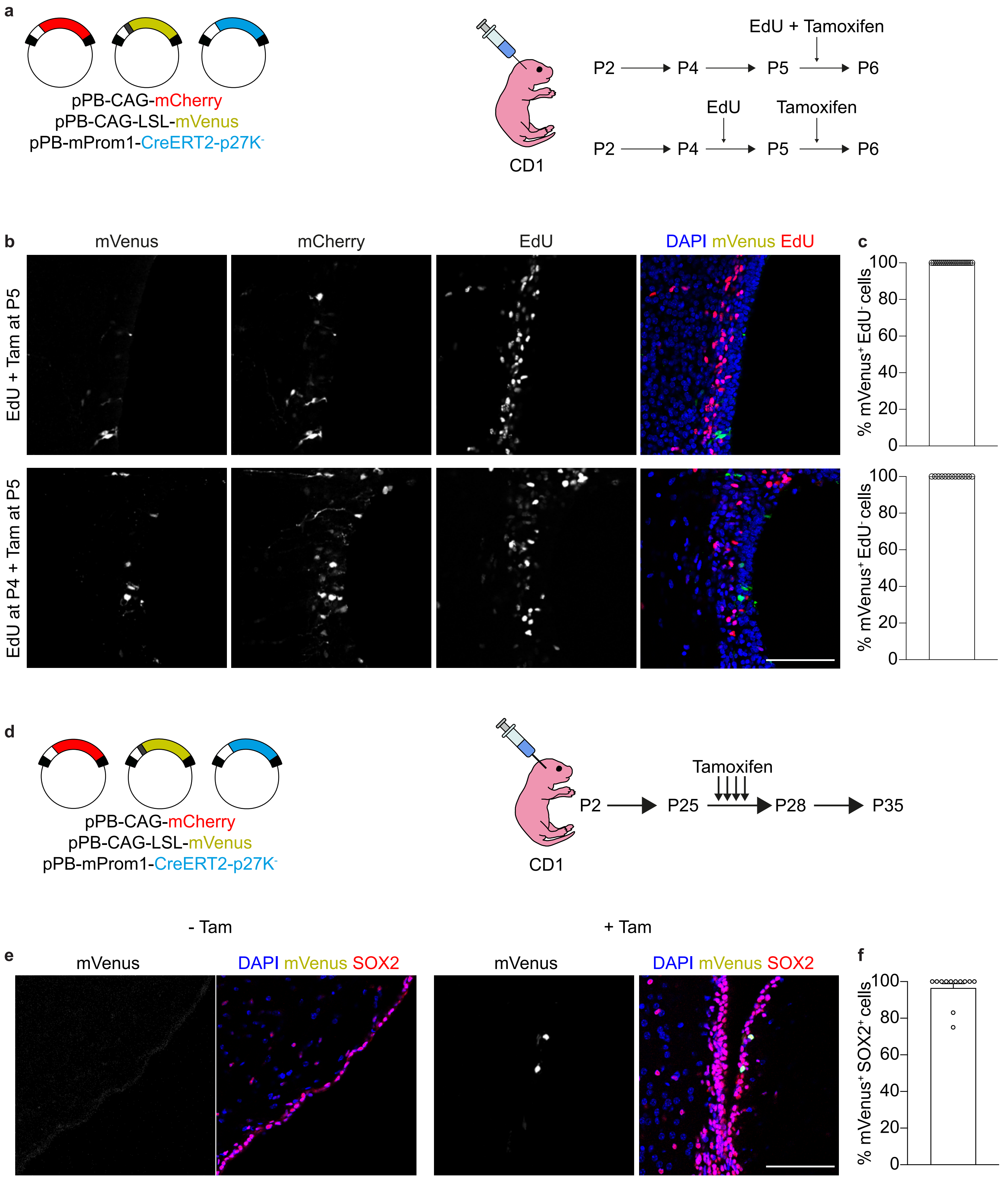

Supplementary Figure 5. Related to Figure 3. Test of the cell cycle specificity of CreERT2-p27K$^-$ in mouse brain. (**a**) Electroporation of cells in the SVZ of P2 CD1 mice with pPB-CAG-mCherry, pPB-CAG-LSL-mVenus and pPB-mProm1-CreERT2-p27K$^-$. Mice were injected with EdU + Tamoxifen at P5 or with EdU at P4 followed by Tamoxifen at P5. All brains were dissected at P6. (**b, c**) Images (**b**) and quantifications (**c**) of mVenus$^+$ cells (green) not labelled with EdU (red) in the SVZ of mice expressing Prom1-CreER-p27 and pPB-CAG-LSL-mVenus and injected with EdU + Tamoxifen at P5 (*n* = 4 mice, 127 cells) or EdU at P4 and Tamoxifen at P5 (*n* = 3 mice, 173 cells). (**d**) Electroporation of cells in the SVZ of P2 CD1 mice with pPB-CAG-mCherry, pPB-CAG-LSL-mVenus and pPB-mProm1-CreERT2-p27K$^-$. Mice were injected daily with Tamoxifen from P25 to P28. All brains were dissected at P35. (**e, f**) Images (**e**) and quantifications (**f**) of mVenus$^+$ cells (green) expressing SOX2 (red) in the SVZ of mice injected with Tamoxifen (*n* = 4 mice, 83 cells). Scale bars (**b, e**) 100 μm. Data are presented as mean ± SEM. Source data are provided as a Source Data file.

Supplementary Figure 6

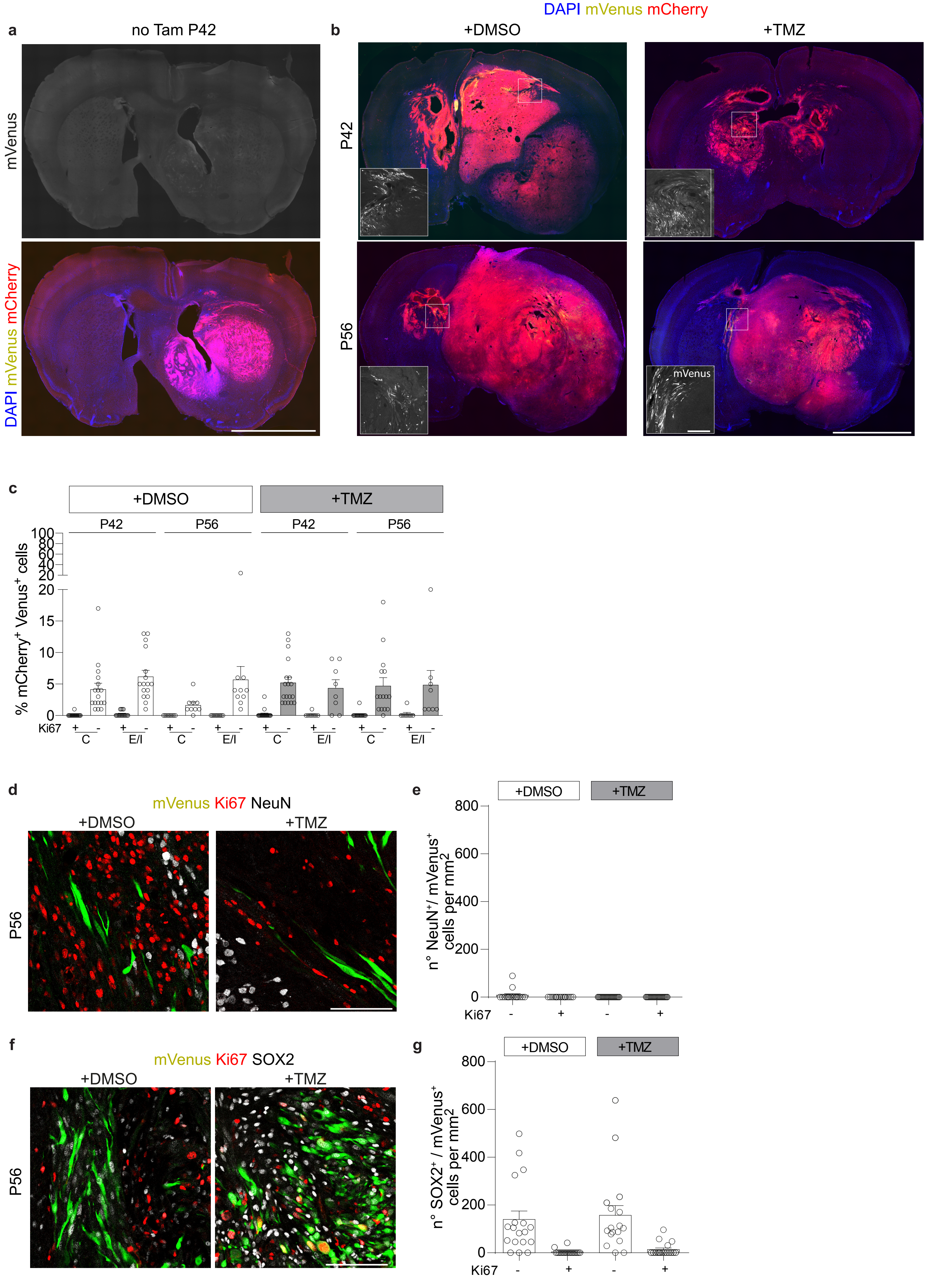

Supplementary Figure 6. Related to Figure 3. Lineage tracing of qProm1 cells in TP-induced tumors after TMZ treatment of mice. (**a**) Images of mVenus$^+$ (green) and mCherry$^+$ (red) cells in brain sections of mice not injected with Tamoxifen at P42 (*n* = 2 mice). (**b**) Images of mVenus$^+$ (green) and mCherry$^+$ (red) in brain sections of DMSO- or TMZ-treated (and Tamoxifen) mice at P42 (*n* = 6 mice for DMSO group; *n* = 7 mice for TMZ group) or P56 (*n* = 4 mice for DMSO group; *n* = 4 mice for TMZ group). (**c**) Quantifications of mCherry$^+$/mVenus$^+$ cells co-expressing or not Ki67 in the core or edge/infiltrative regions of tumors of DMSO- (*n* = 6 mice at P42, 6626 cells for core and 2472 cells for infiltrating edge; *n* = 4 mice at P56, 2679 cells for core and 1298 cells for infiltrating edge) and TMZ-treated (*n* = 7 mice at P42, 4707 cells for core and 1386 cells for infiltrating edge; *n* = 4 mice at P56, 3943 cells for core and 1178 cells for infiltrating edge). (**d, e**) Images (**d**) and quantifications (**e**) of NeuN$^+$ (gray)/mVenus$^+$ (green) cells co-expressing or not Ki67 (red) at P56 in the tumor of mice treated with DMSO (*n* = 2 mice, 70 NeuN$^+$/mCherry$^+$ cells) or TMZ (*n* = 2 mice, 8 NeuN$^+$/mCherry$^+$ cells). (**f, g**) Images (**f**) and quantifications (**g**) of SOX2$^+$ (gray)/mVenus$^+$ (green) cells co-expressing or not Ki67 (red) at P56 in the tumor of mice treated with DMSO (*n* = 2 mice, 1402 SOX2$^+$/mCherry$^+$ cells) or TMZ (*n* = 2 mice, 951 SOX2$^+$/mCherry$^+$ cells). Scale bars (**a, b**) 2 mm and 250 μm (ROI) and (**d, f**) 100 μm. Data are presented as mean ± SEM. Source data are provided as a Source Data file.

Supplementary Figure 7

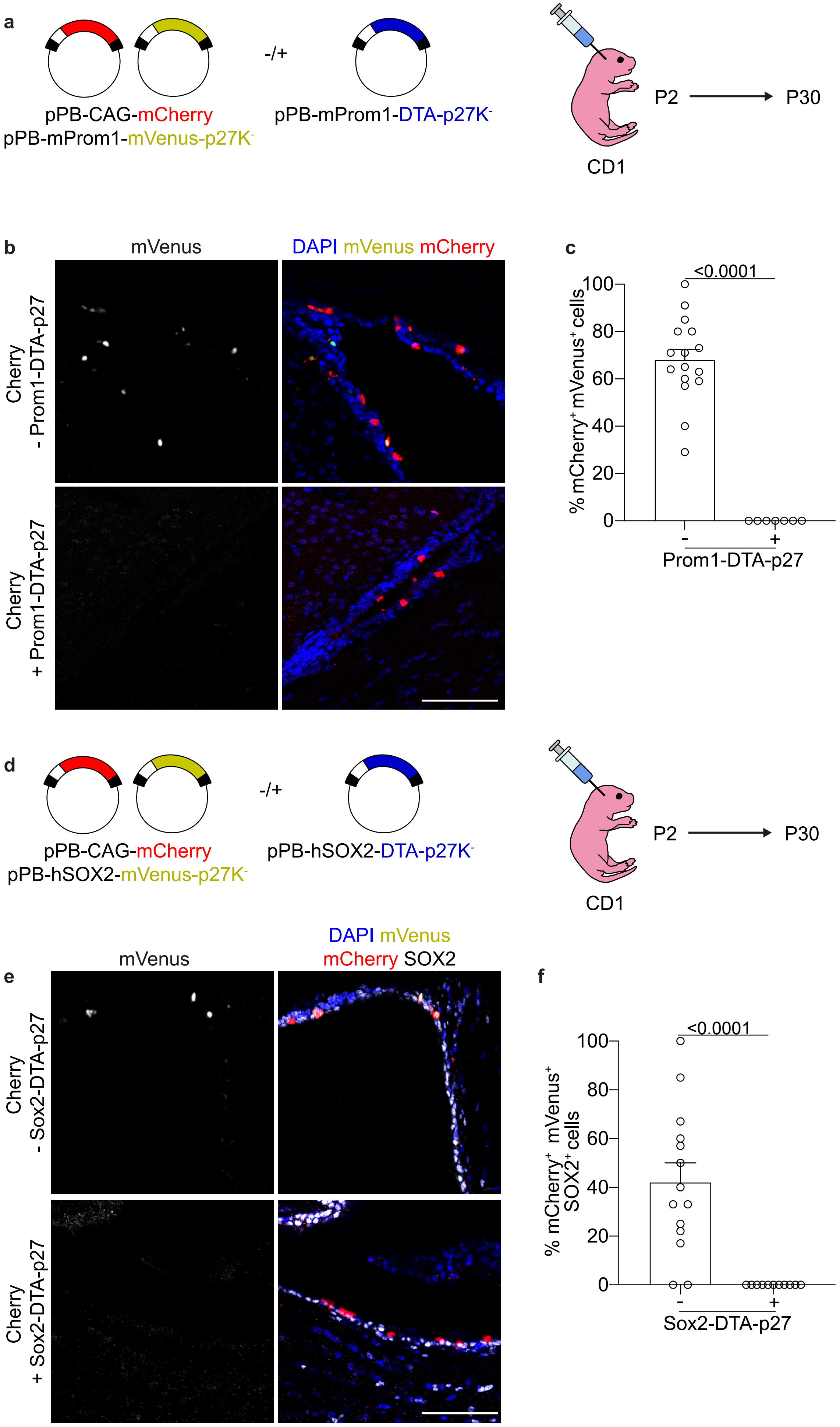

Supplementary Figure 7. Related to Figure 4. Ablation of qProm1 and qSox2 in the SVZ of mouse brain. (**a**) Electroporation of cells in the SVZ of P2 CD1 mice with pPB-CAG-mCherry, pPB-mProm1-mVenus-p27K^{-} with or without pPB-mProm1-DTA-p27K^{-} (Prom1-DTA-p27). Brains were dissected at P30. (**b, c**) Images (**b**) and quantifications (**c**) of mCherry^{+} cells (red) expressing mVenus (green) in the SVZ of mice co-electroporated without (*n* = 4 mice, 267 cells) or with (*n* = 2 mice, 53 cells) Prom1-DTA-p27. (**d**) Electroporation of cells in the SVZ of P2 CD1 mice with pPB-CAG-mCherry, pPB-hSOX2-mVenus-p27K^{-} with or without pPB-hSOX2-DTA-p27K^{-} (Sox2-DTA-p27). Brains were dissected at P30. (**e, f**) Images (**e**) and quantifications (**f**) of mCherry^{+} cells (red) expressing mVenus^{+} (green) and SOX2 (gray) in the SVZ of mice co-electroporated without (*n* = 3 mice, 107 cells) or with (*n* = 3 mice, 74 cells) Sox2-DTA-p27. Scale bars (**b, e**) 100 μm. Data are presented as mean ± SEM. P values were calculated by unpaired two-tailed non-parametric Mann-Whitney test (**c, f**). Source data are provided as a Source Data file.

Supplementary Figure 8

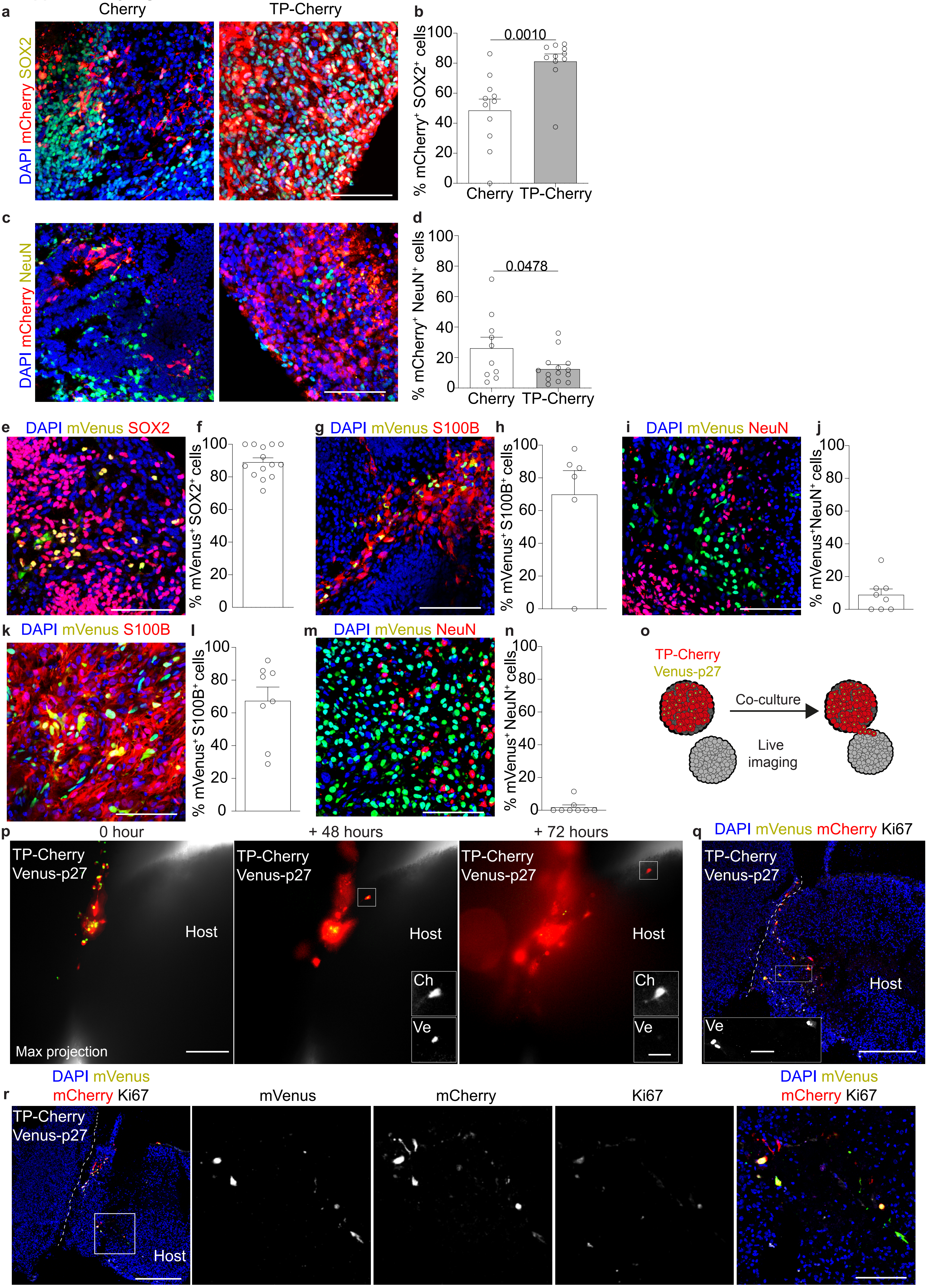

Supplementary Figure 8. Related to Figure 5. Characterization of human brain cancer organoids expressing mVenus-p27K⁻. (**a-d**) Images and quantifications of mCherry^{+} cells (red) co-expressing SOX2 (**a, b**) or NeuN (**c, d**) (green) in organoids at 30 dpe electroporated with Cherry or TP-Cherry (*n* = 4 Cherry organoids, 518 cells for **b;** *n* = 4 Cherry organoids, 424 cells for **d;** *n* = 5 TP-Cherry organoids, 4276 cells for **b;** *n* = 6 TP-Cherry organoids, 5328 cells for **d)**. (**e-j**) Images and quantifications of mVenus^{+} cells (green) co-expressing SOX2 (*n* = 9 organoids, 364 cells) (**e, f**), S100B (*n* = 5 organoids, 210 cells) (**g, h**) or NeuN (*n* = 6 organoids, 265 cells) (**i, j**) (red) in organoids 30 dpe electroporated with TP-Cherry/Venus-p27. (**k-n**) Images and quantifications of mVenus^{+} cells (green) co-expressing S100B (*n* = 2 mice, 641 cells) (**k, l**) or NeuN (*n* = 2 mice, 784 cells) (**m, n**) (red) in TP-Cherry/Venus-p27 organoid grafted in the brain of nude mice. (**o**) Co-culture of TP-Cherry/Venus-p27 at 30 dpe and non-electroporated D60 host organoids. Organoids were imaged with spinning disc confocal microscope every 24h until infiltrating cells were observed (timepoint 0 hour). (**p**) Images of mCherry^{+}/mVenus^{+} cells infiltrating the host. One infiltrating cell is highlighted, and mCherry (Ch) and mVenus (Ve) are separately shown (*n* = 4 co-cultures). (**q,r**) Images of cryosection of the same organoids showed in **p** with infiltrated mVenus^{+} (green)/mCherry^{+} (red)/Ki67^{-} (gray) cells in the host. Scale bars (**a, c, e, g, I, k, m**) 100 μm, (**p**) 200 μm and 50 μm (ROI), (**q, r**) 400 μm and 100 μm (ROI). Data are presented as mean ± SEM. P values were calculated by unpaired two-tailed non-parametric Mann-Whitney test (**b**) or unpaired two-tailed Student t test (**d**). Source data are provided as a Source Data file.

Supplementary Figure 9

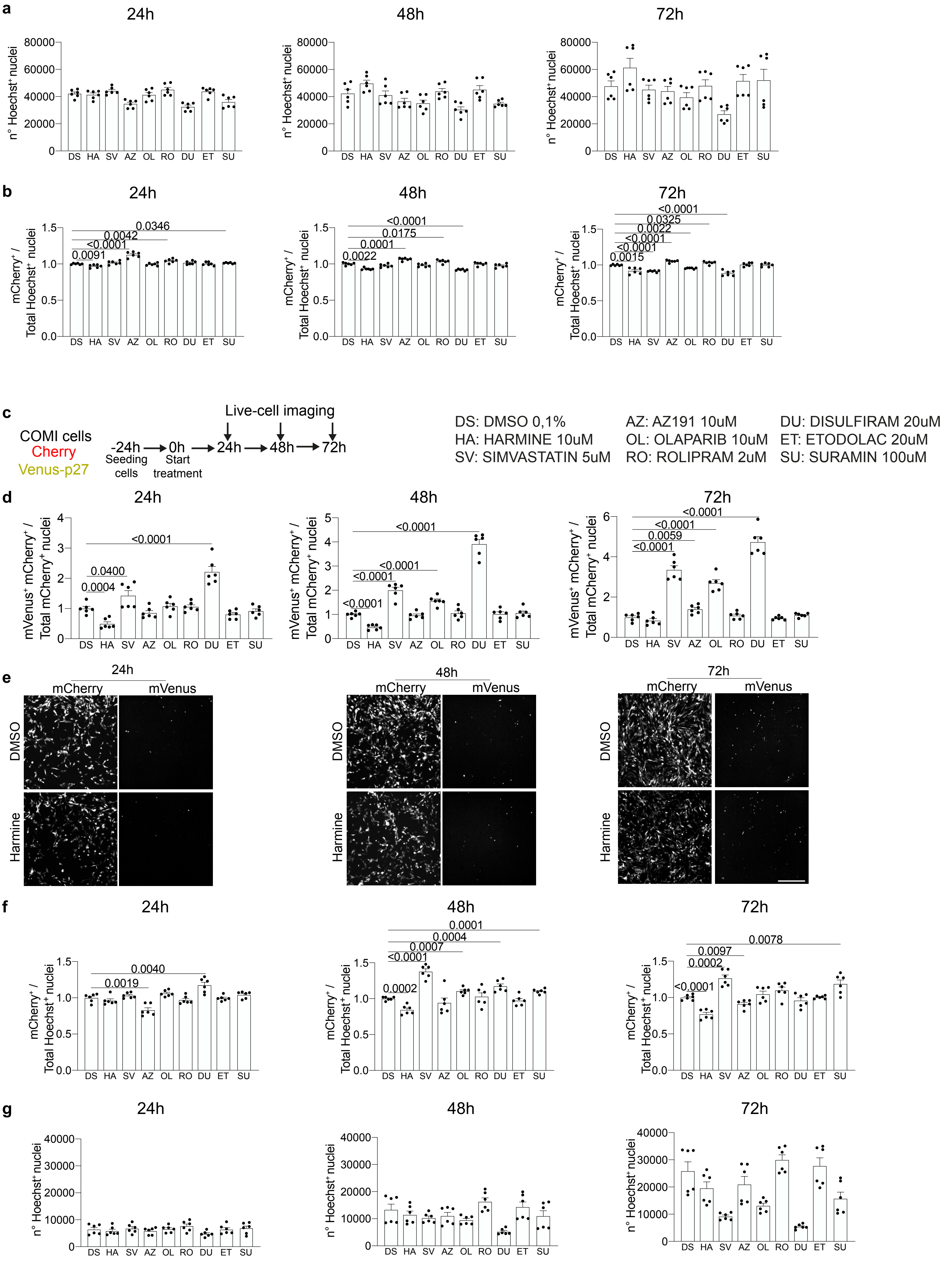

Supplementary Figure 9. Related to Figure 6. Pharmacological targeting of quiescent cells in human GBM cell lines (**a**) Number of Hoechst$^+$ nuclei in GB7 cells at 24h, 48h and 72h post treatment. Each dot represents a well. For each drug 3 wells were imaged, and experiments were conducted in duplicate (*n* = 6 wells per condition). (**b**) Percentage of mCherry$^+$ nuclei over total Hoechst$^+$ nuclei in GB7 cells at 24h, 48h e 72h post treatment. Each dot represents a well (*n* = 6 wells per condition). (**c**) COMI cells previously electroporated with pPB-CAG-mCherry (Cherry) and pPB-CAG-mVenus-p27K$^-$ (Venus-p27) were seeded one day prior treatment. Cells were treated with the listed drugs for 72h and imaged every 24h. Nuclei were counterstained with Hoechst for counting the number of nuclei. For each drug 3 wells were imaged, and experiments were conducted in duplicate. (**d**) Quantification of mVenus$^+$/mCherry$^+$ cells over the total of mCherry$^+$ cells at 24h, 48h and 72h post treatment. Each dot represents a well (*n* = 6 wells per condition). Data were normalized on the DMSO-treated cells on the same day. (**e**) Images of DMSO- or Harmine-treated cells at 24h, 48h and 72h post treatment (*n = 54* images per condition). (**f**) Percentage of mCherry$^+$ nuclei over total Hoechst$^+$ nuclei in COMI cells at 24h, 48h e 72h post treatment. Each dot represents a well (*n* = 6 wells per condition). (**g**) Number of Hoechst$^+$ nuclei in COMI cells at 24h, 48h e 72h post treatment. Each dot represents a well (*n* = 6 wells per condition). Scale bars (**e**) 100 μm. Data are presented as mean ± SEM. P values were calculated by unpaired two-tailed non-parametric Mann-Whitney test or unpaired two-tailed Student t test (**b, d, f**). depending on whether data were normally distributed. Source data are provided as a Source Data file.

Supplementary Figure 10

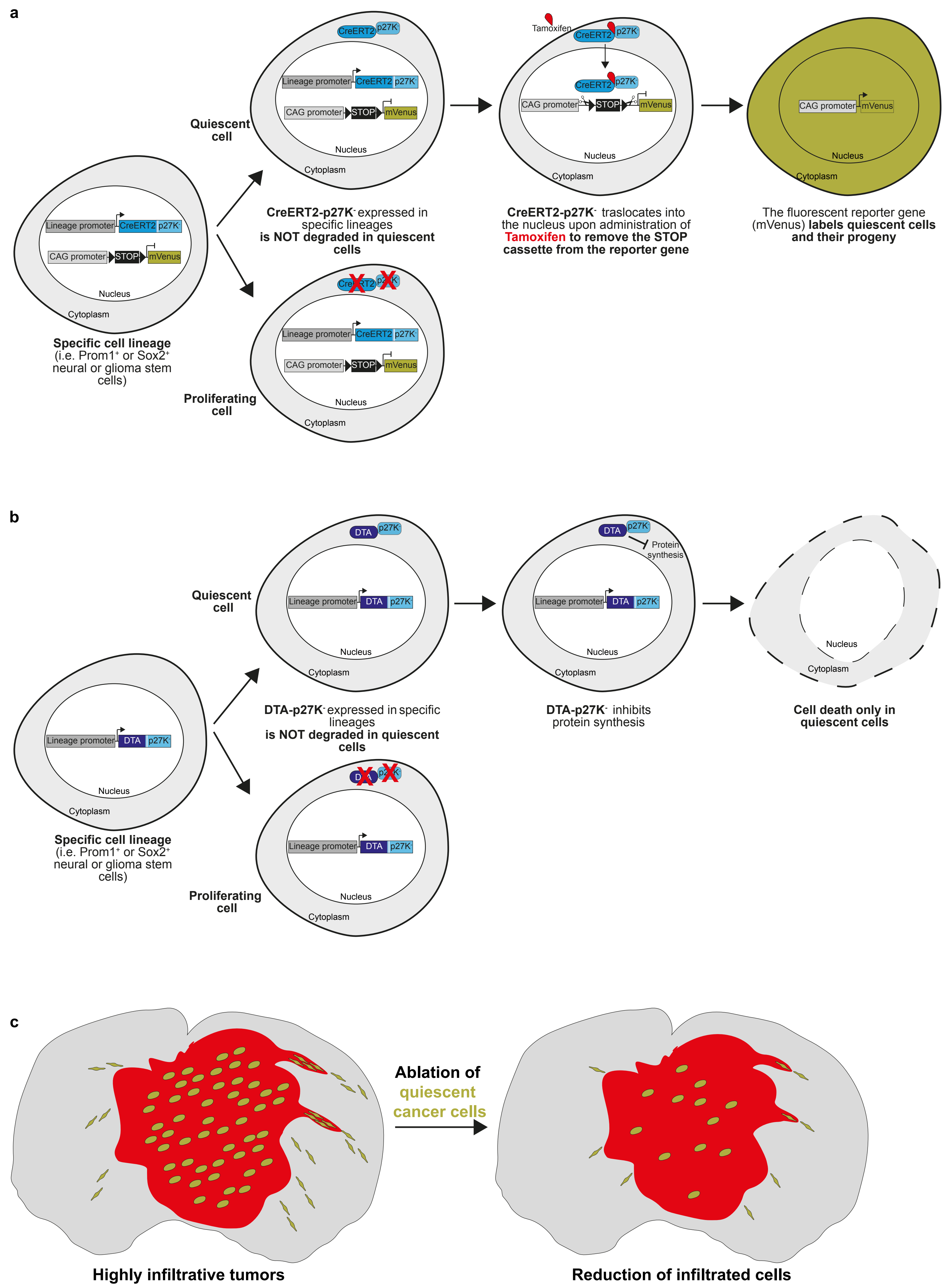

Supplementary Figure 10. Generation of a novel method for CEll cycle-based Lineage Tracing and Ablation of quiescent cells (CELTA). (**a, b**) Schematic view of the method for performing lineage tracing (**a**) and ablation (**b**) in quiescent cells. (**c**) Graphical summary showing the ablation of quiescent cancer cells reduces cancer infiltration.
