# Supplementary material for: A slow-cycling/quiescent cells subpopulation is involved in glioma invasiveness
Source: Nat Commun. 2022 Aug 15;13:4767. doi: 10.1038/s41467-022-32448-0 (PMC9378633; doi:10.1038/s41467-022-32448-0)
Supplement: Supplementary file 2 — Description of Additional Supplementary Files [file 41467_2022_32448_MOESM2_ESM.pdf]

Description of Additional Supplementary Files

File Name: Supplementary Movie 1
Description: **3D projection of infiltrating qProm1 in mouse TP tumors.**
Z-stack of TP-Cherry$^+$ (red)/Prom1-Venus-p27$^+$ (green) expressing cells in the infiltrating edge of TP-induced tumors in mouse brains.

File Name: Supplementary Movie 2
Description: **3D projection of infiltrating quiescent cells derived from TP-Cherry/Venus-p27 organoids transplanted in nude mice brain.**
Z-stack of TP-Cherry$^+$ (red)/CAG-Venus-p27$^+$ (green) expressing cells in the infiltrating edge of TP organoid-derived tumors in mouse brains.

File Name: Supplementary Data 1
Description: differential analysis between pediatric malignant non-cycling PROM1$^+$ cells (PROM1$^+$) and pediatric malignant non-cycling PROM1$^-$ cells (PROM1$^-$).

File Name: Supplementary Data 2
Description: differential analysis between pediatric malignant non-cycling PROM1$^+$ cells (non-cycling) and pediatric malignant cycling PROM1$^+$ cells (cycling).
